# The primary cilium is necessary for the differentiation and the maintenance of human adipose progenitors into myofibroblasts

**DOI:** 10.1038/s41598-017-15649-2

**Published:** 2017-11-10

**Authors:** N. Arrighi, K. Lypovetska, C. Moratal, S. Giorgetti-Peraldi, C. A. Dechesne, C. Dani, P. Peraldi

**Affiliations:** 1 Université Côte d’Azur, CNRS UMR7277, Inserm U1091, IBV, Nice, France; 2 Université Côte d’Azur, Inserm UMR1065, C3M, Nice, France

## Abstract

The primary cilium is an organelle, present at the cell surface, with various biological functions. We, and others, have shown that it plays a role in the differentiation of adipose progenitors (APs) into adipocytes. APs can also differentiate into myofibroblasts when treated with TGF-β1. Several components of the TGF-β1 pathway are located within the cilium suggesting a function for this organelle in AP myofibrogenesis. We studied differentiation of APs into myofibroblasts in two human models: APs of the adipose tissue (aAPs) and APs resident in the skeletal muscles (mAPs). We showed that, *in vivo*, myofibroblasts within muscles of patients with Duchenne Muscular Dystrophy were ciliated. *In vitro*, myofibroblasts derived from APs maintained a functional primary cilium. Using HPI4, a small molecule that inhibits ciliogenesis, and siRNA against Kif-3A, we provide evidence that the primary cilium is necessary both for the differentiation of APs into myofibroblasts and the maintenance of the phenotype. Disruption of the primary cilium inhibited TGF-β1-signalisation providing a molecular mechanism by which the cilium controls myofibroblast differentiation. These data suggest that myofibroblasts from various origins are controlled differently by their primary cilium.

## Introduction

Myofibroblasts appear after tissue lesion and play an active role in tissue repair^1,2^. They are characterized by α-smooth muscle actin (α-SMA) expression and secretion of extracellular matrix (ECM) proteins. When the wound is healed myofibroblasts undergo apoptosis, leaving only a scar. Under some pathological conditions, in most cases associated with inflammation, myofibroblasts persist. This results in an excess of extracellular matrix deposition that can modify the structure of the tissue and affects the homeostasis of the organ, a process called fibrosis^3,4^. In several organs such as lung, liver, heart and kidney, fibrosis causes morbidity and mortality. There is no unique identified precursor of myofibroblasts, but rather, several cells can differentiate into myofibroblasts depending on the organ^1,2^. In the skin, they derive mainly from local fibroblasts. In the kidney, tubular epithelial cells may undergo epithelial-mesenchymal transition to differentiate into myofibroblasts. In pulmonary, cardiac and kidney fibrosis, myofibroblasts can derive from endothelial-mesenchymal transition^5^. Mesenchymal stem cells (MSCs) can also differentiate *in vivo* into myofibroblasts, in cancer for instance^6,7^. Adipocyte progenitors (APs) have been characterized in the adipose tissue (aAPs) and the skeletal muscle (mAPs). aAPs and mAPs (also called FAPs for fibro-adipogenic progenitors) exhibit similar molecular markers and can differentiate into adipocytes and myofibroblasts^8–12^. The differentiation of aAPs and mAPs into myofibroblasts is of particular interest since adipose tissue and muscles are potential sites of fibrosis. Indeed, fibrosis is a prominent pathological feature of skeletal muscles in patients with Duchenne Muscular Dystrophy (DMD) where mAPs have been identified as the cellular source of myofibroblasts^13,14^. Fibrosis also develops within the adipose tissue during obesity, and is responsible, by part, for insulin resistance, a situation that can evolve into diabetes^15–17^. A better understanding of events governing the conversion of APs into myofibroblasts could provide us with new therapeutic targets for the treatment of fibrosis.

TGF-β1 is considered as a master regulator of myofibroblasts differentiation^18^. TGF-β1 is a cytokine that binds two receptors: TGFβR1 (ALK5) and TGFβR2 which are serine/threonine kinases^19^. Upon activation, the TGF receptors phosphorylate Smad2 and Smad3. These proteins form heterotrimers with Smad4 and translocate into the nucleus where they control the transcription of target genes. TGF-β1 can also stimulate a non-canonical pathway involving TGF-β activated kinase 1, phosphatidylinositol 3-kinase, AKT and Rho-like GTPase signaling pathways. Both canonical and non-canonical pathways appear necessary for myofibroblasts differentiation^18^. TGFβR1 and R2 as well as Smad2/3 and Smad4 have been located in the primary cilium. Moreover, cells with a stunted cilium are less sensitive to TGF-β1-induced Smad phosphorylation^20^. However, the role of the primary cilium in mediating myofibroblast differentiation of APs remained to be studied.

The primary cilium is an antenna-shaped organelle present in most cells of the organisms characterized by acetylated alpha tubulin (Ac-Tub) concentrated in the axoneme^21–23^. The cilium has various functions. It is used as a mechanoreceptor in kidney cells and bears the photoreceptors in rods and cones. It is also involved in the transduction pathway of several molecules such as Hedgehog and Wnt. Thus, it plays an important role in embryogenesis and cell differentiation. Mutations causing a total loss of the primary cilium are embryonic lethal and mutations of proteins involved in the homeostasis of the cilium are responsible for ciliopathies. Ciliopathies are rare diseases (around 1 in 1000 births) associated with disparate disorders^24,25^. For instance, Bardet Biedl syndrome is associated, among other traits, with obesity. We, and others, have shown that the primary cilium plays a role in adipocyte differentiation of aAPs^26–31^.

Epithelium mesenchymal transition of LLC-PK1 into myofibroblast is associated with a loss of the cilium and its signaling activities. In addition, inhibition of the primary cilium in these cells decreased the expression of α-SMA, a marker of myofibroblasts^32^. Here, we have investigated if the cilium was involved in the differentiation of mesenchymal human aAPs and mAPs into myofibroblasts after TGF-β1 treatment. *In situ*, myofibroblasts from muscles of patients suffering from Duchenne muscular dystrophy (DMD) were ciliated. We present evidence that TGF-β1 induced differentiation of these APs into myofibroblasts without a loss, but a shortening of the cilium. Cilia of myofibroblasts were functional since cells responded to Hedgehog. The primary cilium appeared necessary both for the differentiation of AP into myofibroblasts but also for the maintenance of the differentiated phenotype. Mechanistically, loss of the cilium was associated with a decrease in TGF-β1 induced Smad phosphorylation. Together, these data pointed to a crucial function of the primary cilium in the differentiation of APs into myofibroblasts and suggest that myofibroblasts from different origins behave differently.

## Results

### The primary cilium is shortened but maintained a signaling activity during differentiation of APs into myofibroblasts

**Figure 1:**
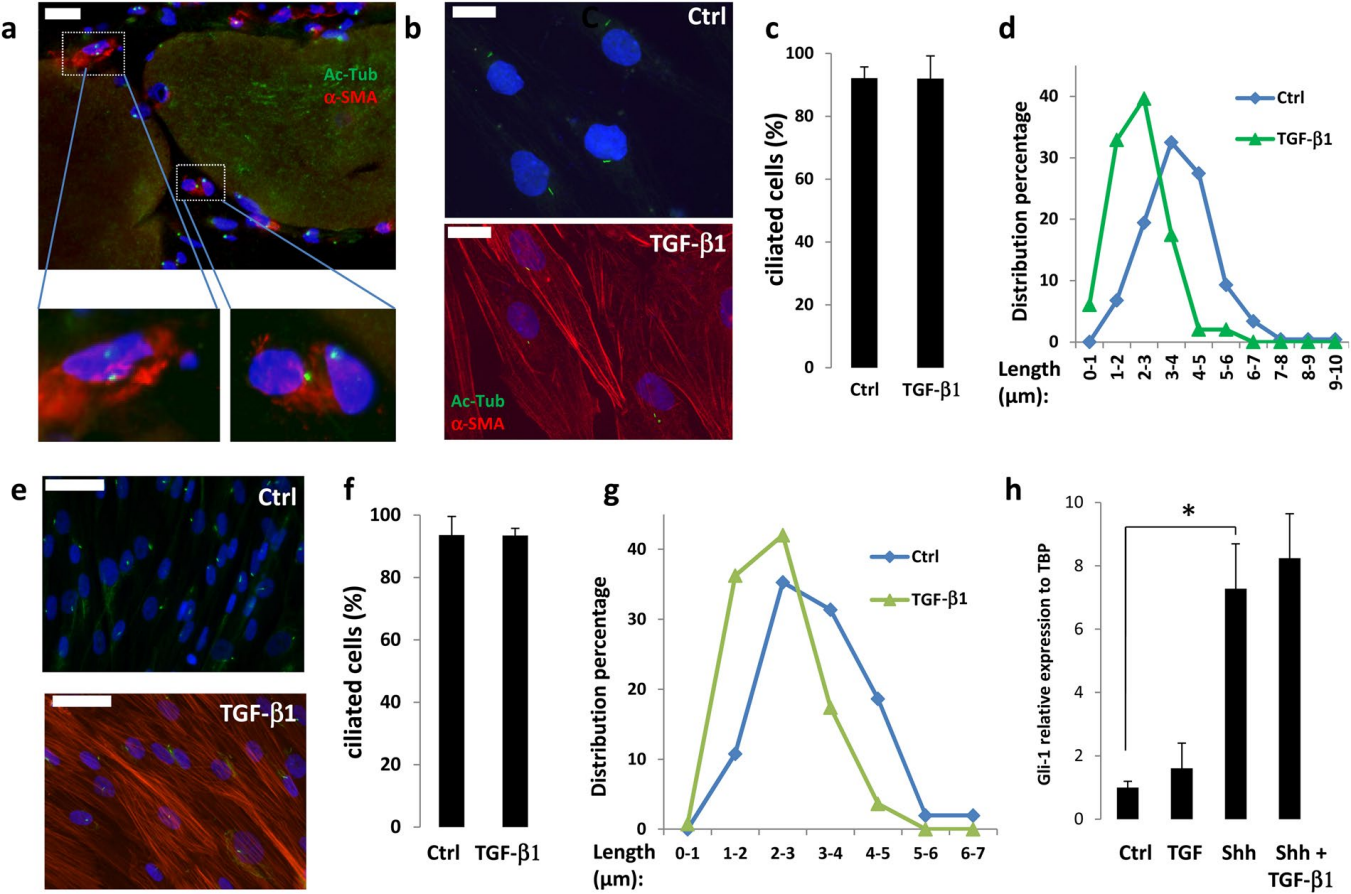
(**a**) Sections of skeletal muscle from DMD patients were fixed. Ac-Tub (in green) and α-SMA (red) were revealed by immunocytochemistry, nuclei were stained with Hoechst 33258 (blue). The white bar represents 20 μm. (**b**) mAPs (white bars: 20 μm) or e) aAPs (white bars: 50 μm) were treated or not with 5 ng/ml of TGF-β1. Ac-Tub (green) and α-SMA (red) were revealed by immunocytochemistry. (**c**) mAPs or F) aAPs cilia were counted and compared to the number of nuclei. (**d**) mAPs or G) aAPs distribution of cilia lengths of one representative experiment. (**h**) aAPs were treated with TGF-β1  for 3 days then with Shh for 24 h. Gli-1 expression was assessed using quantitative RT-PCR (mean + /− sd of a representative experiment (*p < 0.05 n = 3)).

We investigated if myofibroblasts were ciliated *in vivo* using muscle of DMD patients as a model of fibrotic tissue^13,14^. The primary cilium was visualized through acetylated alpha tubulin (Ac-Tub) labeling^21^. As expected, the DMD muscle was disorganized and fibers were surrounded by fibrillary collagen, visualized by second-harmonic imaging microscopy (Supplementary Fig. 1). Most of the cells in the inter myofiber space were ciliated. Ac-Tub, and α-SMA labeling revealed that myofibroblasts localized in fibrotic area of DMD muscles patients were ciliated (Fig. 1a). Several ciliated cells were present, but no myofibroblast was detected in healthy muscles (Supplementary Fig. 2).

We previously showed that during early adipocyte differentiation of aAPs, the primary cilium increases in length then disappears in differentiated adipocytes^33,34^. We analyzed the size of the primary cilium during myofibroblast differentiation of mAPs (Fig. 1b–d) and aAPs (Fig. 1e–g). TGF-β1 treatment induced an increase in α-SMA-labelled cells (Fig. 1b and e, an enlargement of the pictures is presented in Supplementary Fig. 3). Undifferentiated APs and TGF-β1-treated cells were nearly all ciliated indicating that myofibroblasts maintained a primary cilium (Fig. 1c and f). The identity of the primary cilium was ascertained in aAP and myofibroblasts from aAP by double labeling with antibodies to acetylated tubulin, that labels the axoneme of the cilium, and pericentrin, a marker of the centrioles of the basal body, located at the base of the primary cilium^35^ and ARL13B which localized to the ciliary membrane^36^, and pericentrin (Supplementary Fig. 4). Then, we measured the length of the cilium as described in^33^ (Fig. 1d and g). APs myofibroblast differentiation was associated with a shortening of the cilium (from 3.72 μm + /−0.19 to 2.54 μm + /− 0.14 p < 0.005 for mAPs and 3.3 μm + /− 0.16 to 2.47 μm + /− 0.12 p < 0.001 for aAPs).

In chondrocytes, TGF-β1 decreases IFT88 expression, inducing a shortening of the cilium^37^. We tested the effect of TGF-β1 on the expression of several genes associated with ciliogenesis or cilium resorption: IFT88, HDAC-6, KIF-3a and NEDD9 in aAPs (Supplemental Fig. 5). None of them was significantly modified. Although we cannot rule out that post-transcriptional modifications of these genes could be involved in the mechanism studied, these results indicated that the mechanism of cilia shortening in myofibroblasts was different than the one described in chondrocytes.

We analyzed if the decrease in the cilium size was associated with a decrease of its signaling activity. To do this, we studied the response of the cells to Sonic Hedgehog (Shh) which signaling pathway depends upon the cilium. aAPs were treated with or without TGF-β1 for 3 days to convert them into myofibroblasts. Cells were then treated with Shh-conditioned medium for 24 h. RNA were extracted and Gli-1 expression, a reliable marker of Shh signaling^38–40^, was analysed by quantitative RT-PCR (Fig. 1h). Shh induced a similar increase in Gli-1 expression in undifferentiated aAPs and in myofibroblasts. Together these results indicated that myofibroblasts had a functional primary cilium.

### The primary cilium controls myofibroblast differentiation

**Figure 2:**
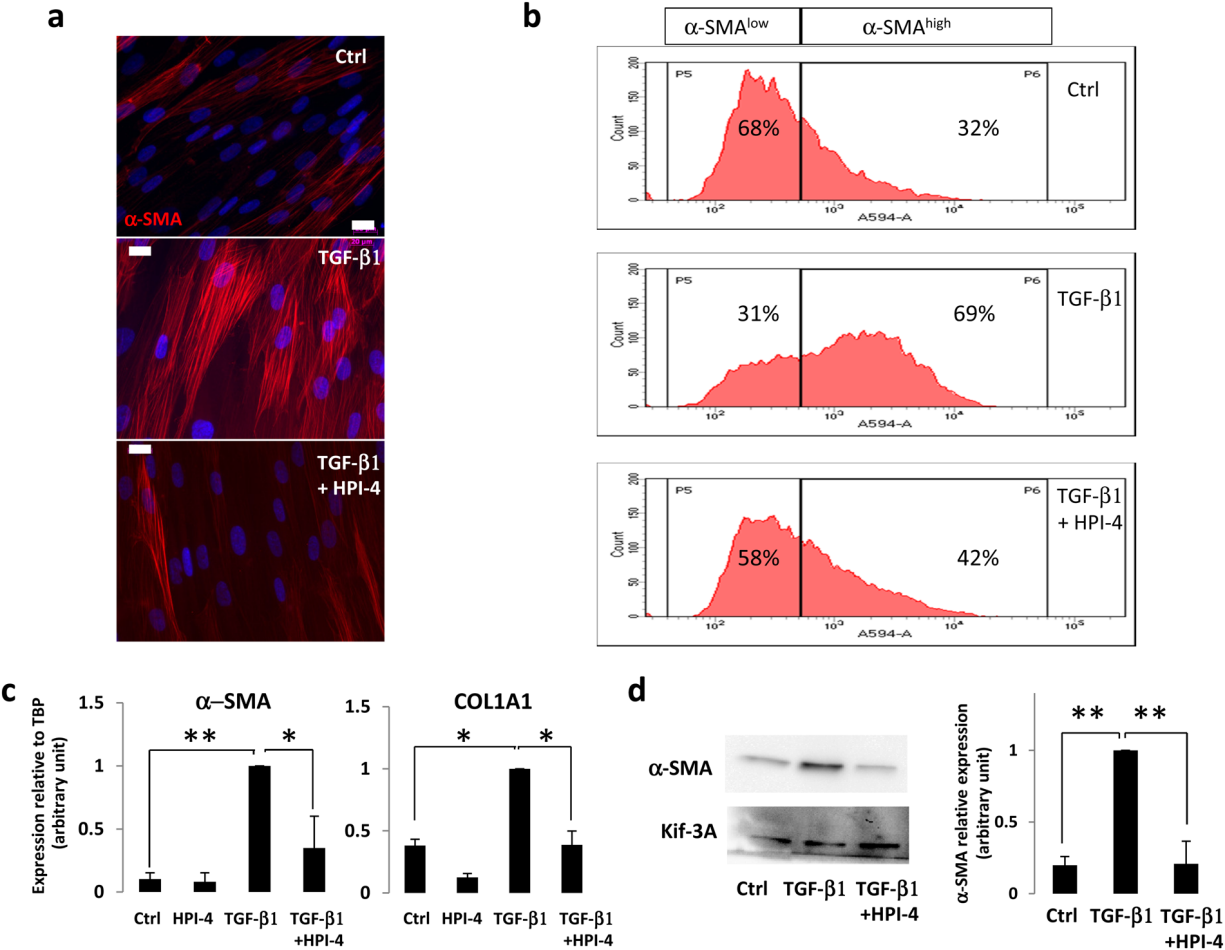
(**a**) aAPs were treated for 24 h with HPI-4 then HPI-4 was removed and TGF-β1 added for 72 h. α-SMA (red) was revealed by immunocytochemistry, nuclei were stained with Hoechst 33258^57^ (white bar: 20 μm) (**b**) Cells treated as described in A were sorted by FACS for α-SMA expression. The percentages of α-SMA^low^ and α-SMA^high^ are indicated. (**c**) aAPs were treated as indicated and quantitative RT-PCR were performed. (n = 3 *p < 0.05. **p < 0.01) D) Cells were treated as indicated and Western blot performed using the indicated antibodies. A quantification of 4 experiments is provided (**p < 0.001).

To determine the function of the primary cilium during myofibroblast differentiation of APs we used HPI-4, a ciliogenesis inhibitor^41^. HPI-4, also known as ciliobrevin A, is a well described inhibitor of the cilium formation. It is a specific inhibitor of dynein *in vitro* and in cell culture^42^. Inhibition of dynein function leads to abnormal protein trafficking within the primary cilium followed by a loss of the cilium. A 24 h HPI-4 treatment of aAPs led to an 88% decrease in the number of ciliated cells (Supplementary Fig. 6). We tested the effect of the cilium loss on myofibroblast differentiation. aAPs were treated for 24 hours with HPI-4. HPI-4 was removed and cells were treated for 3 days with TGF-β1. Pretreatment of aAPs with HPI-4 induced a decrease in myofibroblast differentiation as visualized by α-SMA labelling (Fig. 2a). We evaluated the percentage of myofibroblast differentiation inhibition at the cellular level through FACS analysis. The results revealed a α-SMA^low^ population (undifferentiated cells) and a α-SMA^high^ population (myofibroblasts) that increased upon TGF-β1 treatment (Fig. 2b, a dot plot representation is provided in Supplementary Fig. 7). When cells were pretreated with HPI-4 the ratio of myofibroblasts sharply decreased. Using Q-RTPCR we tested the expression of two myofibroblast markers; α-SMA and COL1A1 (Fig. 2c). α-SMA protein levels were also analyzed by Western blot (Fig. 2d). Both methods revealed that the loss of the cilium was associated with an inhibition of the expression of myofibroblast markers. Similar results were obtained with mAPs (Supplementary Fig. 7).

**Figure 3:**
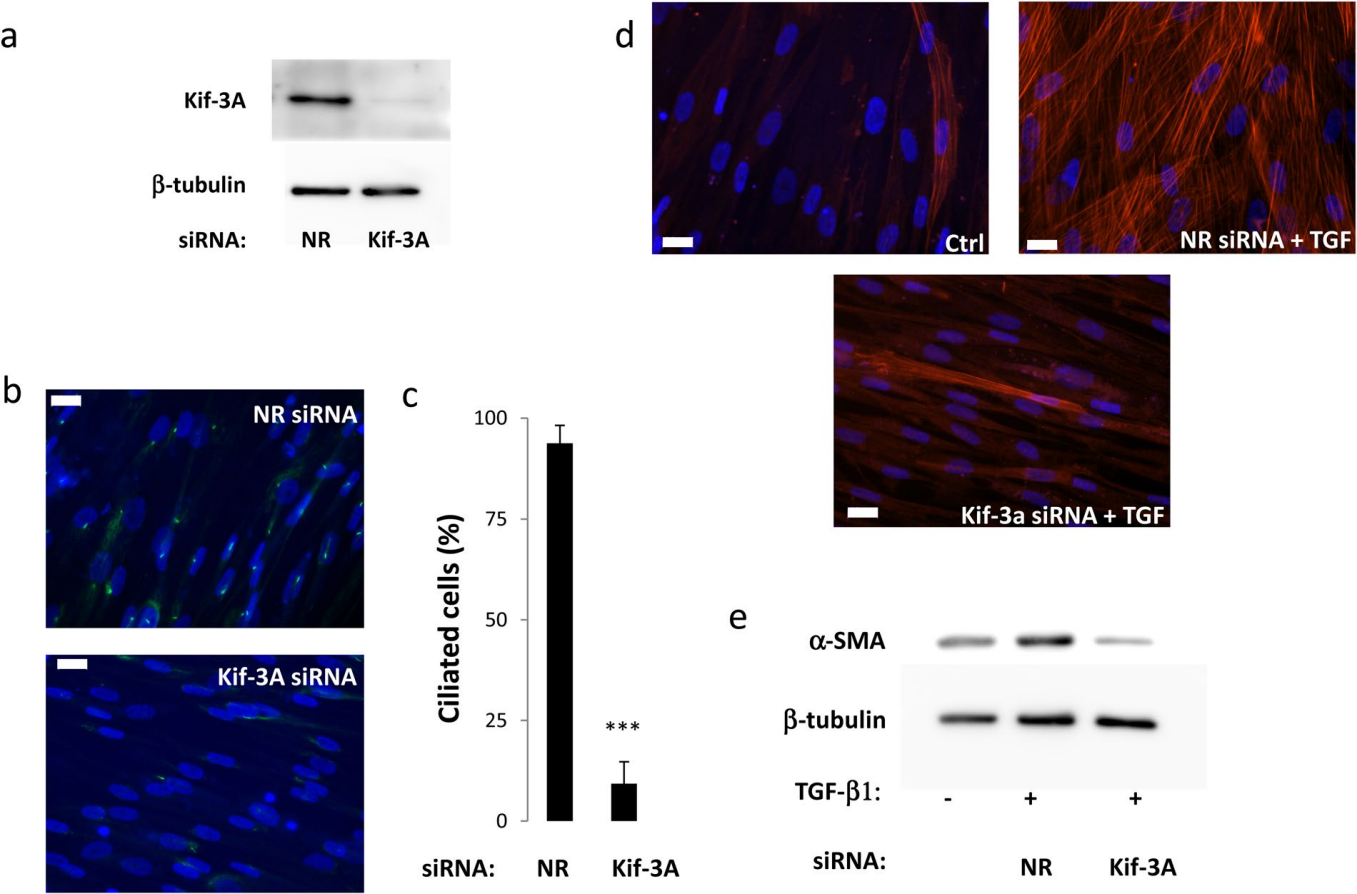
(**a**) aAPs were treated with a non-relevant (NR) or a Kif-3A siRNA as described in Material and Methods. (**a**) Kif-3A expression was analyzed by Western blot. (**b**) Cells were fixed and Ac-Tub (in green) was revealed by immunocytochemistry. The white bar represents 20 μm. (**c**) Percentages of ciliated cells were measured (n = 3 ***p < 0.001). Cells were treated or not with TGF-β1 for 3 days and α-SMA expression was analyzed by (**d**) immunofluorescence or (**e**) Western blot.

In order to confirm these results, we used a siRNA approach to destabilize the primary cilium. KIF-3A is a subunit of kinesin-2, the expression of which has been shown to be necessary for the primary cilium^21,22^. KIF-3a siRNA induced an inhibition of Kif-3A protein expression and led to a deciliation of aAP (Fig. 3a,b and c). Deciliation of aAP by KIF-3A siRNA decreased the ability of TGF-β1 to induce myofibroblast differentiation of the cells as visualized by α-SMA labelling of the cells (Fig. 3d) and by a 71 + /− 12% decreased in α-SMA protein levels assessed by Western blot (Fig. 3e).

These results indicated that the primary cilium is necessary for the differentiation of APs into myofibroblasts.

**Figure 4:**
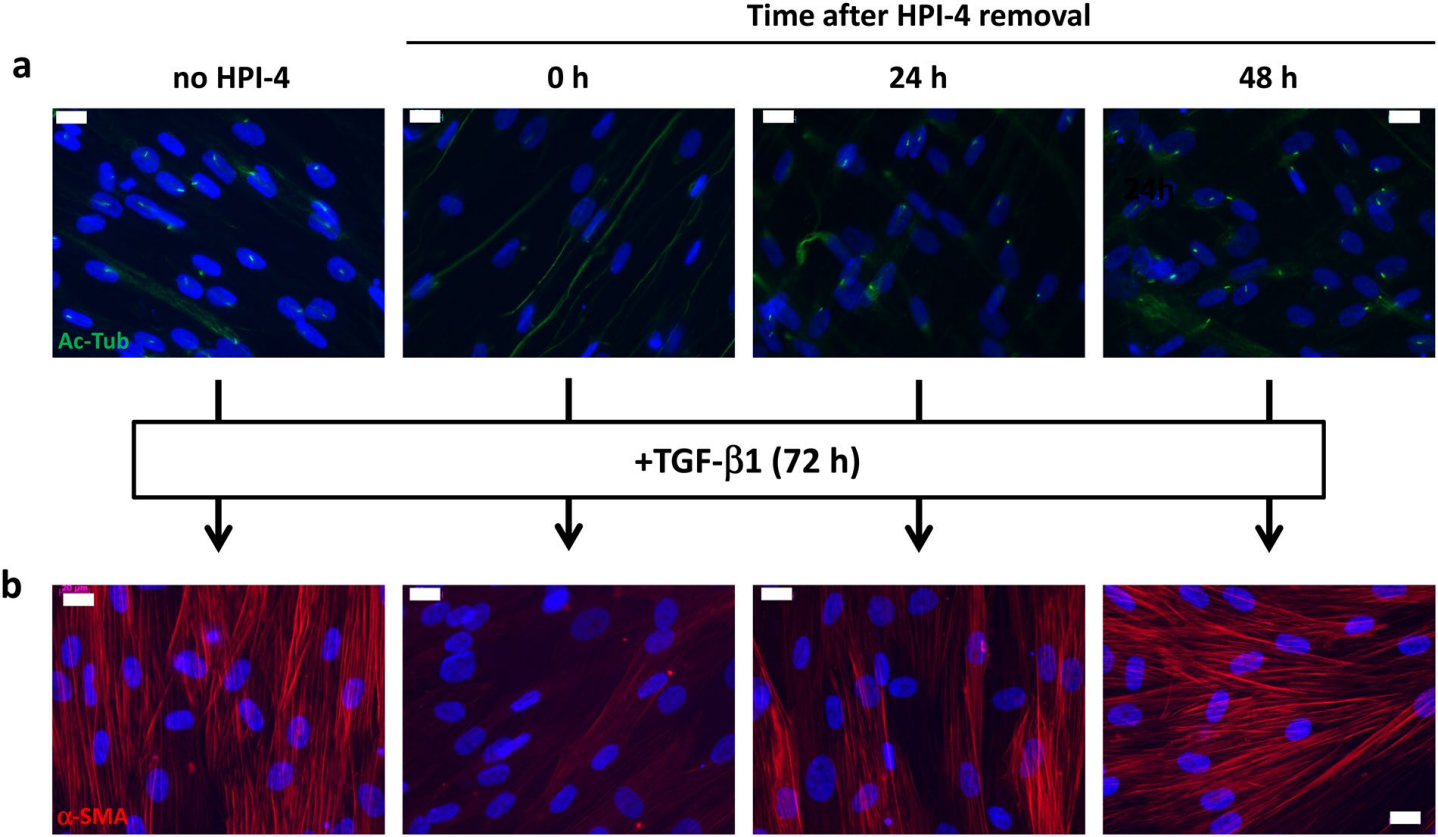
(**a**) aAPs were treated with or without HPI-4 for 24 h. Immediately after (0 h), 24 h or 48 h after HPI-4 removal cells were fixed and Ac-Tub (in green) was revealed by immunocytochemistry. (**b**) Cells treated as described in A were treated for three days with TGF-β1. α-SMA (in red) was revealed by immunocytochemistry. The white bar represents 20 μm.

It has been described that the effect of HPI-4 is reversible^42^. We investigated the kinetics of recovery of the cilium after HPI-4 treatment and determined if the deletion of the primary cilium irreversibly inhibited the ability of aAPs to differentiate into myofibroblasts. aAPs were incubated or not with HPI-4 for 24 h. Then, HPI-4 was removed from the culture medium and the primary cilium was visualized by immunofluorescence after 0 h, 24 h or 48 h (Fig. 4a). Cells lost their cilium after HPI-4 treatment and began to recover it 24 h after HPI-4 removal. The recovery was total after 48 h. We analyzed the ability of TGF-β1 to induce myofibroblasts differentiation after these treatments (Fig. 4b). As observed previously, just after HPI-4 treatment cells lost their capacity to undergo myofibrogenesis. When TGF-β1 was applied 24 h after the removal of HPI-4 most of the cells differentiated into myofibroblasts and, after 48 h, myofibroblast differentiation was similar to the condition where cells were never exposed to HPI-4. Thus, the ability of the cells to recover their myofibroblast differentiation properties occurred simultaneously with the recovery of the cilium.

Together, these data indicate that the primary cilium was necessary for TGF-β1 induced differentiation of APs into myofibroblasts.

### The primary cilium is necessary for the maintenance of the myofibroblast phenotype

**Figure 5:**
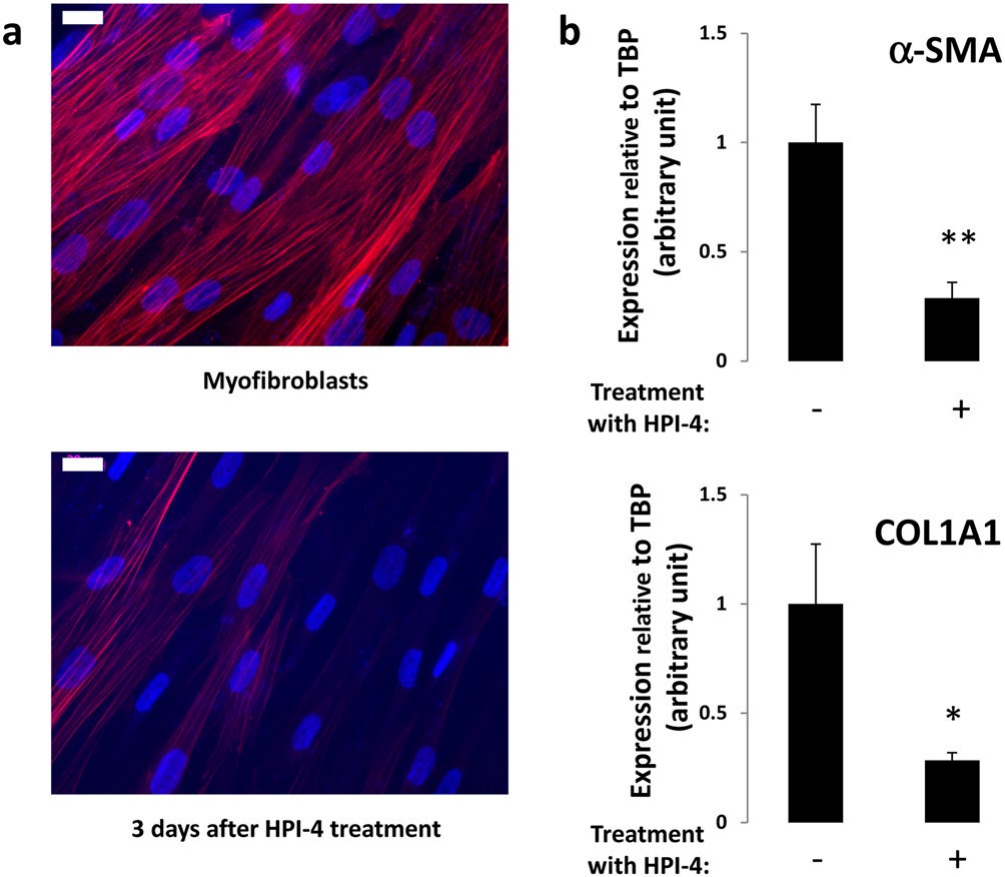
aAP were treated for 3 days with TGF-β1 then with or without HPI-4 for 24 h. HPI-4 was removed and, after 3 days, (**a**) cells were fixed and α-SMA was revealed by immunocytochemistry and (**b**) RNA were extracted and α-SMA and COL1A1 expression was assessed using quantitative RT-PCR (mean + /− sd of a representative experiment (*p < 0.05 **p < 0.01 n = 3).

Since myofibroblasts differentiated from AP possess a primary cilium we investigated the function of this organelle in the maintenance of the myofibroblast phenotype. Confluent aAP were treated for 3 days with TGF-β1 to induce myofibroblast differentiation then with or without HPI-4 for 24 h. After 3 days we observed that deletion of the cilium led to a decrease in myofibroblast differentiation as analyzed by immunofluorescence using α-SMA antibodies (Fig. 5a) and by the expression of α-SMA and COL1A1 (Fig. 5b). These data indicated that the primary cilium was necessary for the maintenance of the myofibroblast phenotype.

### The primary cilium is necessary for TGF-β1-induced Smad phosphorylation

**Figure 6:**
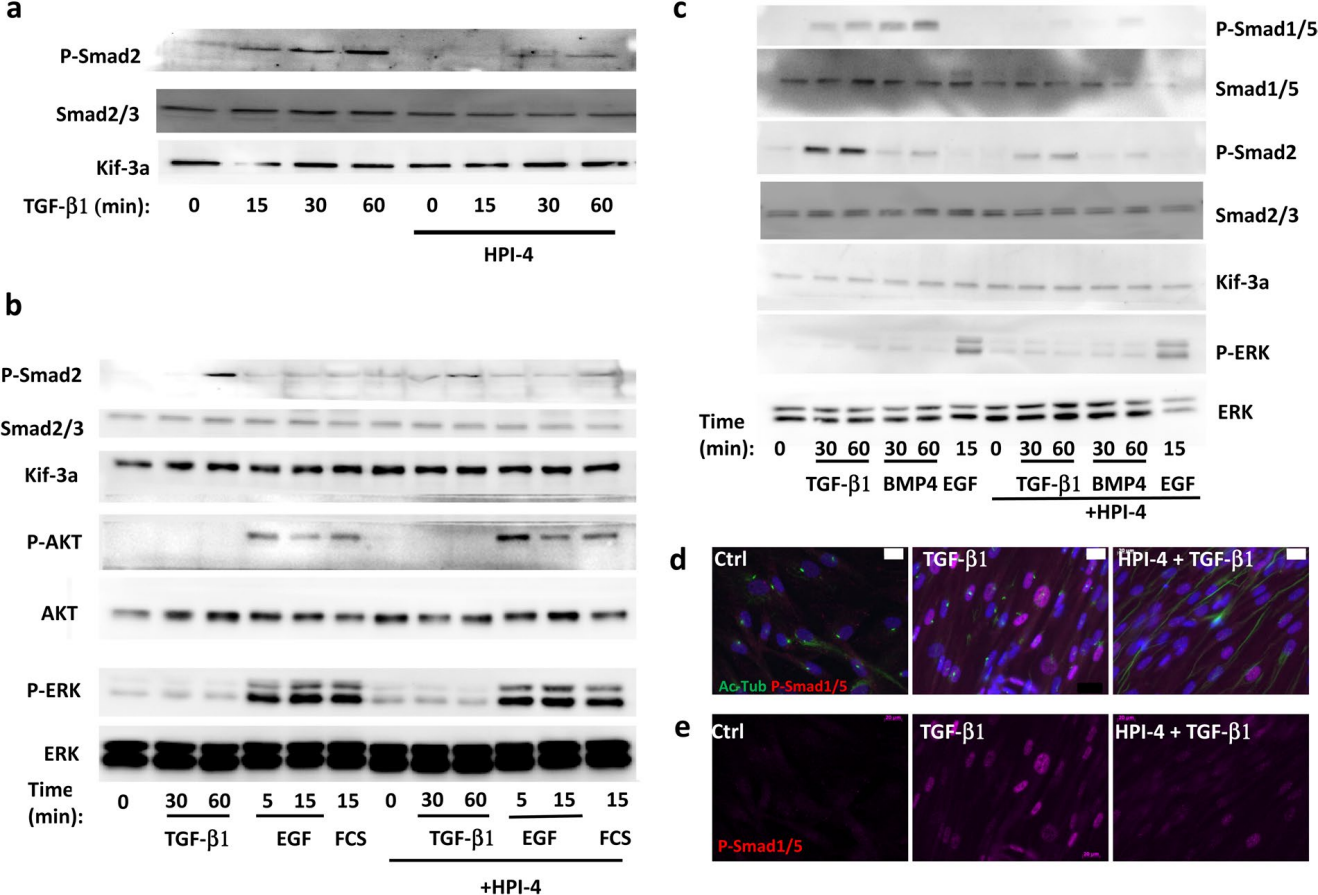
(**a**–**c**) aAPs were treated as indicated and Western blots performed using the indicated antibodies. “(**d**) After a 24 h HPI-4 treatment cells were treated for 1 h with TGF-β1. Ac-Tub (in green) and phospho Smad1/5 (red) were revealed by immunocytochemistry. The white bar represents 20 μm. (**e**) The phospho Smad1/5 (red) channel from (**d**) is presented.

We investigated the molecular mechanisms by which HPI-4 inhibited myofibroblast differentiation. TGF-β1 mediates its signaling through the phosphorylation of Smad proteins^19^. Part of this signaling pathway is located within the primary cilium^20^. aAPs were treated for 24 h with HPI-4 then for 15, 30 or 60 min with TGF-β1. Cells were lysed and Smad2 phosphorylation was analyzed by Western blot. Smad2 expression was analyzed using antibodies against Smad2/3 and Kif-3a was used as a protein loading control (Fig. 6a). TGF-β1 induced an increased in Smad2 phosphorylation which was maximal after 1 hour. In cells treated with HPI-4 this phosphorylation was decreased by 74% + /− 19 (p < 0.01, n = 4). We studied if the effect of HPI-4 was specific to cilia-associated signaling. aAPs were treated for various times with TGF-β1, EGF or fetal calf serum (FCS) in cells pre-treated or not with HPI-4 (Fig. 6b). HPI-4 inhibited TGF-β1 induced Smad2 phosphorylation but did not affect AKT or ERK phosphorylation induced by EGF and FCS.

We studied if the phosphorylation of other Smads could be inhibited. In particular, we investigated BMP4 signaling since one of its receptor, BMPR2, has been located in the cilium^20^. Cells were treated with TGF-β1, BMP4 and EGF as a control and the phosphorylation of Smad1–5 and Smad2 were analyzed by Western blot (Fig. 6c). TGF-β1 and BMP4 increased Smad1/5 and Smad2 phosphorylation. HPI-4 inhibited the phosphorylation of Smad1/5 by 90% in TGF-β1-treated cells and 95% in BMP4-treated cells. Smad2 phosphorylation inhibition was respectively 65% and 50% in TGF-β1  and BMP-4 treated cells.

We analyzed the cellular localization of active/phosphorylated Smad1/5. Cells were pretreated or not with HPI-4 then treated with TGF-β1. Phospho-Smad1/5 (red) and Ac-Tub were revealed by immunochemistry (Fig. 6d and e). For a better visualization, only the phospho-Smad1/5 channel is presented in Fig. 6e. In untreated cells, no phospho-Smad1/5 was detected. TGF-β1  induced an increase in phospho-Smad1/5 within the nucleus. Cells pretreated with HPI-4 lost their primary cilium and TGF-β1-induced Smad1/5 phosphorylation was inhibited.

Together these results demonstrated that inhibition of ciliogenesis leads to an inhibition of TGF-β1 signaling.

## Discussion

The primary cilium has emerged as a novel organelle involved in several differentiation processes, in particular in adipocytes, neuronal cells and in cells from the skin^22,43^. Here, we present evidence that the primary cilium is necessary for the acquisition and the maintenance of a myofibroblast phenotype of AP under TGF-β1 treatment.

Myofibroblasts are cells expressing α-SMA and producing ECM proteins that are abundant in fibrosis^1,2^. Myofibroblast is not a classically well-defined cell type derived from one progenitor but rather can be produced from various cell types after various stimuli. Indeed, myofibroblasts are produced from different cells depending on the organ and the injury. As such, it is unlikely that all myofibroblasts are the product of the same differentiation program. The proportion of which type of cells is responsible for the production of myofibroblasts in each situation of fibrosis is still unclear. MSCs have been proposed to be the prominent precursors of myofibroblasts in some cancers and in the corneal stroma after irregular phototherapeutic keratectomy^6,7^. aAPs and mAPs have a similar ability to differentiate into adipocytes and myofibroblasts^8–12^. Our data indicate that they also both depend upon the primary cilium to convert into myofibroblasts.

These observations unveil a new level of heterogeneity between myofibroblasts derived from different cell type. Both epithelial–myofibroblast (EMT) transition of kidney cells into myofibroblast^32^ and myofibroblast differentiation of AP through TGF-β1 treatment are dependent upon the primary cilium. However, while differentiation of kidney cells into myofibroblast is associated with a loss of the cilium and a decrease of its associated signaling properties^32^, myofibroblasts differentiated from AP maintained a shorter, but functional primary cilium which appeared necessary to maintain the phenotype. It could be interesting to determine if the shortening of the cilium in AP is mechanistically identical to the first step of the primary cilium loss observed in the differentiation of kidney cells. This mechanism could be responsible for the generation of two populations of myofibroblasts. Myofibroblasts derived from AP could still be responsive to signaling pathway depending of the cilium (such as Hh, Wnt, TGF-β1) and be dependent of a cilium to maintain their phenotype while myofibroblasts derived from kidney cells would not^32^.

Because of their location, the study of APs is particularly relevant in the context of fibrosis. Indeed, during obesity the adipose tissue become hypoxic and suffers a state of chronic mild inflammation associated with an increase in circulating TGF-β1 and the development of fibrosis. Together, these events are responsible for the development of an insulin resistant state that can evolve into diabetes^15,16^. In the muscles of DMD patients a state of chronic inflammation develops and the muscles are invaded by adipocytes and myofibroblasts. The structure of the muscle is disorganized resulting in muscular weakness^13,14^.

Here we showed that, in aAPs and mAPs, TGF-β1-induced myofibroblast differentiation was associated with a decrease of the primary cilium length. TGF-β1 has been shown to decrease the primary cilium length in chondrocytes^37^. However, the mechanisms in both cell types appear different since we did not observe any modification in IFT88 expression, as was observed in chondrocytes. So far, the reason of the decrease in length is unknown. Since cell contractility has been demonstrated to regulate ciliogenesis in RPE1 cells^44^, it could be hypothesized that the mechanical tension caused by stress fibers in myofibroblasts creates constraints that limited the cilium length. *In vitro*, this decrease in length did not modify the response of the cells to Shh. Since the surface of the primary cilium in myofibroblasts would be smaller it cannot be excluded that, *in vivo*, a decrease in the primary cilium length could lead to a diminished sensitivity of the cells to circulating factors. Thus, myofibroblasts from AP can still respond to factors the signaling pathways of which depend upon the cilium. In addition, the cilium could be involved in the association between myofibroblasts and the ECM during fibrosis^45^. Indeed, integrin receptors, present in the cilium, allow the binding of the cells to collagen fibers. It has been shown to be of particular interest during bone extension where chondrocytes interact with the ECM through integrin and NG2 chondroitin sulfate proteoglycan localized within the primary cilium. This allows an appropriate orientation of chondrocytes leading to tissue anisotropy. Association of myofibroblasts with the ECM through the primary cilium could have an important biological function.

We also demonstrate that the primary cilium of myofibroblasts is necessary to maintain the differentiated phenotype. Maintenance of the phenotype of myofibroblasts from lung fibroblasts is dependent upon autocrine secretion of TGF-β1^46^. It could be hypothesized that deletion of the primary cilium in myofibroblast from AP inhibits the level of TGF-β1  signaling that is necessary to maintain a differentiated phenotype. It can be noted that, at this point, we cannot totally rule out that the effect of HPI-4 on the maintenance of the phenotype is linked to an effect on dynein, independent of the cilium.

The involvement of the cilium in fibrosis *in vivo* is suggested by the observation that ciliopathies are associated with massive fibrosis^22,24,47^. However, so far it has not been unambiguously demonstrated that the development of fibrosis is a direct effect of ciliopathies. *In vitro*, we showed that the primary cilium was necessary for the differentiation of APs into myofibroblast. There is an apparent discrepancy between the *in vitro* data, where the transient inhibition of ciliogenesis inhibits myofibroblast differentiation and the observation that ciliopathies are associated with increased fibrosis. In this regard, it is interesting to note that a total loss of the cilium results in fetal death while ciliopathies are associated with dysfunction of the cilium. In several cases the cilium is still present^48^. Thus, the effect of a malfunction of the cilium differs from the one caused by a total loss of the cilium. For instance, a total deletion of the cilium *in vitro* leads to an inhibition of adipocyte differentiation^27,29^. By contrast, mutation of BBS proteins in ciliopathies is associated with obesity and a better adipocyte differentiation *in vitro* ^26,30^. This phenomenon is reminiscent of Shh, a morphogen dependent upon the primary cilium, that exerts different effects according to its concentration^49^.

We also proposed a mechanism that allows the cilium to control myofibroblast differentiation. Deletion of the primary cilium inhibited the ability of TGF-β1 to stimulate Smads phosphorylation. Since Smads play a central role in myofibroblast differentiation, inhibition of their phosphorylation could be sufficient to explain the decrease in differentiation^2,18^. TGF-β1  receptor and Smad have been reported to be located in the primary cilium^20^. Thus, disorganization of the cilium structure that concentrates TGF-β1  signaling resulted in an inhibition of its signaling pathway. It could be noted that TGF-β1 is functional in cells that do not possess a primary cilium such as adipocytes and hepatocytes. Consequently, it cannot be assumed that all cells that possess a primary cilium are dependent upon this organelle for TGF-β1 signaling. This suggest that, depending on the cell context, the TGF-β1  signaling pathway has a different location and thus probably a different functioning. For instance, in aAPs, TGF- β1 did not activate AKT and ERK (Fig. 4) while it does in hepatocytes.

Deletion of the primary cilium was also associated with an inhibition of Smad1/5 phosphorylation by TGF-β1  and BMP4. TGF-β1 has already been shown to increase the phosphorylation of Smad1/5^50^. Thus, in addition to the TGF-β1  pathway, the primary cilium appeared important for BMP4 signaling in aAPs. This could have interesting consequences since BMP4 signaling plays a role in osteoblast and chondrocyte differentiation^51^, two cell types that can be derived from aAPs.

It has been proposed to treat fibrosis through inhibition of Shh, TGF-β1  or PDGF-R signaling^4^. These three pathways are dependent to some extent upon the primary cilium. This has been demonstrated for Shh. It has been shown that the PDGF receptor, which is present in the cilium, is necessary for mAPs differentiation into myofibroblasts^52^. Here, we present evidence that the primary cilium was involved in TGF-β1  mediated myofibroblast differentiation of APs. Thus, the primary cilium controls three fibrotic signaling pathways. Since TGF-β1  has important anti-inflammatory activity, inhibitors of TGF-β1 for the treatment of fibrosis could be counterproductive. However, as stated above, TGF-β1  signaling is not dependent upon the primary cilium in all cell types. Thus, therapeutic approaches that would target the primary cilium at the region of fibrosis could prove beneficial especially since deciliation would inhibit myofibroblast differentiation and induce the dedifferentiation of myofibroblasts. The potential of the primary cilium as a therapeutic target has been underscore by the study of Kopinke *et al*.^53^. They showed that deletion of the cilium of mAP in a mouse model of dystrophic mice decreases intramuscular adipogenesis, enhances myogenesis and increases myofiber size. Although the authors have not studied myofibroblasts in the muscle of these mice, fibrosis is a prominent pathological feature of skeletal muscles in patients with DMD. It could be interesting to determine if the improvement of the muscle physiology of these dystrophic mice is also associated with a decrease in fibrosis.

Together, our study demonstrates a new function of the primary cilium for the acquisition and the maintenance of a myofibroblast phenotype after TGF-β1  treatment of AP. Moreover, we unveil the existence of myofibroblasts with different signaling properties.

## Material and Methods

### Material

Antibodies monoclonal against Ac-Tub and α-SMA were from Sigma-Aldrich (Saint Quentin Fallavier, France), rabbit against Kif-3A was from Abcam (Cambridge, UK) rabbit anti-AKT, phospho-Akt (thr 308), Smad, phospho-Smad, ERK, phospho-ERK were from Cell Signaling (Ozyme, St Quentin en Yvelines, France), monoclonal anti ARL13B (sc-515784) was from Santa Cruz (Ozyme, St Quentin en Yvelines, France), rabbit anti pericentrin was from Bethyl (Euromedex, Souffleweyrsheim, France). Antibody against mouse coupled to Alexa Fluor 488 and antibody against rabbit coupled to Alexa fluor 647 were from Life Technologies (Saint Aubin, France). Antibodies against CD56 APC and CD140a PE were purchased from BD-Biosciences (Le Pont de Claix, France). HPI-4 was from Sigma-Aldrich. Shh-conditioned medium was obtained from an HEK 293 cell line stably transfected with Shh-N expression vector (ATCC #CRL-2782).

### Cell culture

#### aAPs

Establishment of human aAPs have been previously described^54^. aAPs were grown in Dulbecco’s modified Eagle’s medium (DMEM) supplemented with 10% fetal calf serum (FCS), 2.5 ng/ml hFGF-2, 60 μg/ml penicillin, and 50 μg/ml streptomycin. At day 2 post-confluence (designated day 0), cells were maintained in DMEM with 10 µg/ml transferrin. TGF-β1  (5 ng/ml) was added for three days. HPI-4 was used at 60 μM.

#### mAPs and tissue section

All human samples were obtained with the inform consent of the donors. Tissue sections were obtained from *res nullus* from surgeries performed on healthy donors with the approval of the Centre Hospitalier Universitaire de Nice Review Board, according to the rules of the French Regulatory Health Authorities. DMD and age-matching control biopsies were obtained from Myobank-AFM Institut de Myology, Paris, France. 5 μm cryostat sections from paravertebral muscles were performed on biopsies from a 15 years old male. mAPs were prepared by enzymatic digestion of muscle samples as previously described^12^. Cells were grown as adherent cells in Ham’s F10, 20% fetal bovine serum, 10 mM Hepes, 2,5 ng/ml basic fibroblast growth factor 2, 1 µM dexamethasone, 100 U/ml penicillin, and 100 mg/ml streptomycin. Progenitors were sorted at passage 2 or 3 by flow cytometry as CD140a (PDGFRα)-positive cells and CD56-negative for mAPs. Cell separations were performed using a BD FACSARIA II sorter with the BD FacsDiva software as previously described^12^. Efficiency of sorting was checked after 2 or 3 passages after cell sorting. Cells were used until passage 10. Myofibroblast differentiation was induced after confluence in the same medium complemented with 5 ng/ml TGF-β1 for 5 days.

#### siRNA

aAP were seeded at 18000 cells /cm². 24 hours after they were transfected with a siRNA directed against Kif-3A (s21942, ThermoFisher scientific, Villebon-sur-Yvette, France,) or with a non-relevant siRNA using HiPerfect Transfection Reagent (Qiagen, Courtaboeuf, France) as recommended by the manufacturer. The transfection was replicated 3 days later and experiments performed after another 3 days.

#### Immunocytochemistry

Cells were seeded on glass coverslips and treated as described in the text. Cells were rinsed with PBS and fixed with Roti-Histofix (Roth, Lauterbourg, France) for 20 min at room temperature. Fixed-cells were incubated in PBS with 3% bovine serum albumin (BSA), 0.1% tween-20 and 0.1% triton X-100 for 30 min at room temperature. Cells were incubated with the appropriate antibodies in the same buffer for 90 min at room temperature. After 3 washes in PBS, coverslips were incubated with the appropriate secondary antibody coupled to AlexaFluor (1:600) for 45 min at room temperature. When α-SMA and Ac-Tub were used together, α-SMA antibody was covalently linked to Alexa Fluor 595 using the Zenon mouse IgG labelling kits (molecular probes, Invitrogen) according to the manufacturer instructions. Cells or sections were mounted in mowiol. Images were taken on an Axio Observer microscope (Carl Zeiss, Le Pecq, France) with an EC Plan Neofluar 40X (NA 1.3) or a Plan Apochromat 63X (NA 1.4) oil objective using AxioVision 4.8.2 software. At least ten representative fields were examined for each condition. The total number of cilia was counted along with total number of nuclei for at least 80 cells. Cilia length was measured using Fiji^55^ as described in^56^.

#### Second Harmonic Generation (SHG) imaging

Imaging was performed on a LSM 780 NLO inverted Axio Observer.Z1 confocal microscope (Carl Zeiss Microscopy GmbH, Jena, Germany) using a Plan Apo 25X multi immersion (oil, glycerol, water) NA 0.8 objective. The SHG light source was a Mai Tai DeepSee (Newport Corp., Irvine, CA, USA) tuned at 880 nm. Forward SHG signal was detected with Oil condenser (1.4 NA), bandpass filter 440/40 nm and transmission PMT. Backward SHG was collected on GaAsP (BIG) non descanned module with 440/10 nm.

#### Western Blot

Cells were washed with ice-cold phosphate-buffered saline (6 mM Na_2_HPO_4_, 1 mM KH_2_PO_4_, pH 7.4, 140 mM NaCl, 3 mM KCl) and lysed in RIPA buffer (50 mM Tris pH 7.5, 150 mM NaCl, 1% NP40, 0.1% SDS, 0.5% Na Deoxycholate, 1 mM Orthovanadate, 5 mM NaF, 2.5 mM Na_4_P_2_O_7_ and Complete Protease Inhibitor Cocktail (Roche Diagnostics, Meylan, France) for 20 min at 4 °C. Lysates were centrifuged (8000 g) for 10 min. 10 μg of proteins were resolved on SDS-PAGE under reducing conditions on a 10% polyacrylamide gel. Proteins were transferred to a polyvinylidene difluoride membrane (Merck Millipore, Fontenay sous Bois), and Western blot analysis were revealed using a Biorad Chemidoc XRS + imaging system. Full-length Western blots are provided in the supplementary data.

#### FACS analysis

aAPs were plated on 100 mm dishes. After treatment cells were trypsinised, washed with PBS and incubated with Roti-Histofix (Roth, Lauterbourg, France) for 20 min at room temperature. Fixed cells were incubated in PBS with 3% bovine serum albumin (BSA), and 0.2% triton X-100 for 20 min at room temperature. Cells were incubated with α-SMA antibody in the same buffer for 30 min at room temperature then with a secondary antibody coupled to Alexa Fluor 594 for 30 min at room temperature. Cells were analysed with BD LSR Fortessa and FACS DIVA Software (Becton Dickinson) on the basis of 10^4^ events.

#### RNA extraction and analysis

Total RNAs were extracted with the TRI-Reagent kit (Euromedex, Souffelweyersheim, France) according to manufacturer’s instructions. Total RNA was subjected to real-time quantitative reverse transcription (RT)-polymerase chain reaction (PCR) analysis as described in^31^. Primers were designed using Primer Express software (Applied Biosystems, Courtaboeuf, France) and validated by testing PCR efficiency using standard curves (85% ≤ efficiency ≤ 115%). Gene expression was quantified using the comparative C_T_ (threshold cycle) method on a StepOnePlus system (Applied Biosystems); TBP was used as reference. The sequences of the primers used are provided in the supplementary table.

#### Statistics

Data are shown as means +/− SD. Statistically differences between groups were analyzed using Student’s t test or ANOVA using graphPad InStat 3.02.

## Electronic supplementary material

Supplementary figures
