# Supplementary material for: The primary cilium is necessary for the differentiation and the maintenance of human adipose progenitors into myofibroblasts
Source: Sci Rep. 2017 Nov 10;7:15248. doi: 10.1038/s41598-017-15649-2 (PMC5681559; doi:10.1038/s41598-017-15649-2)

Supplementary Information

The primary cilium is necessary for the differentiation and the maintenance of human adipose progenitors into myofibroblasts

Arrighi N, Lypovetska K, Moratal C, Giorgetti-Peraldi S, Dechesne CA, Dani C, Peraldi P.

	**Forward**	**Reverse**
**TBP**	CACGAACCACGGCACTGATT	TTTTCTTGCTGCCAGTCTGGAC
**Gli1**	TGCAGTAAAGCCTTCAGCAATG	TTTTCGCAGCGAGCTAGGAT
**α-SMA**	TGCCTGATGGGCAAGTGA	CTGGGCAGCGGAAACG
**COL1A1**	ACCTGCGTGTACCCCACTCA	CCGCCATACTCGAACTGGAA
**FN1**	CTGGCCGAAAATACATTGTAAA	CCACAGTCGGGTCAGGAG
**HDAC6**	CCCCGCTCTATCCCCAAT	CGAGCTTCTTCATTTTGCCTTT
**Kif3A**	TACTGGACAGCGCCTAAAGGA	CCAAGGCAGAAATTACATTACCAA
**IFT88**	TGACATCTGCAAAACTCATTGCT	TCCACGCACCAATCATAACCT
**Nedd9**	GAGACCCATTTCATTTCCCTTCT	GGGCTGAGCTGACACAACTG

Supplementary Table: sequence of primers used for RT-QPCR

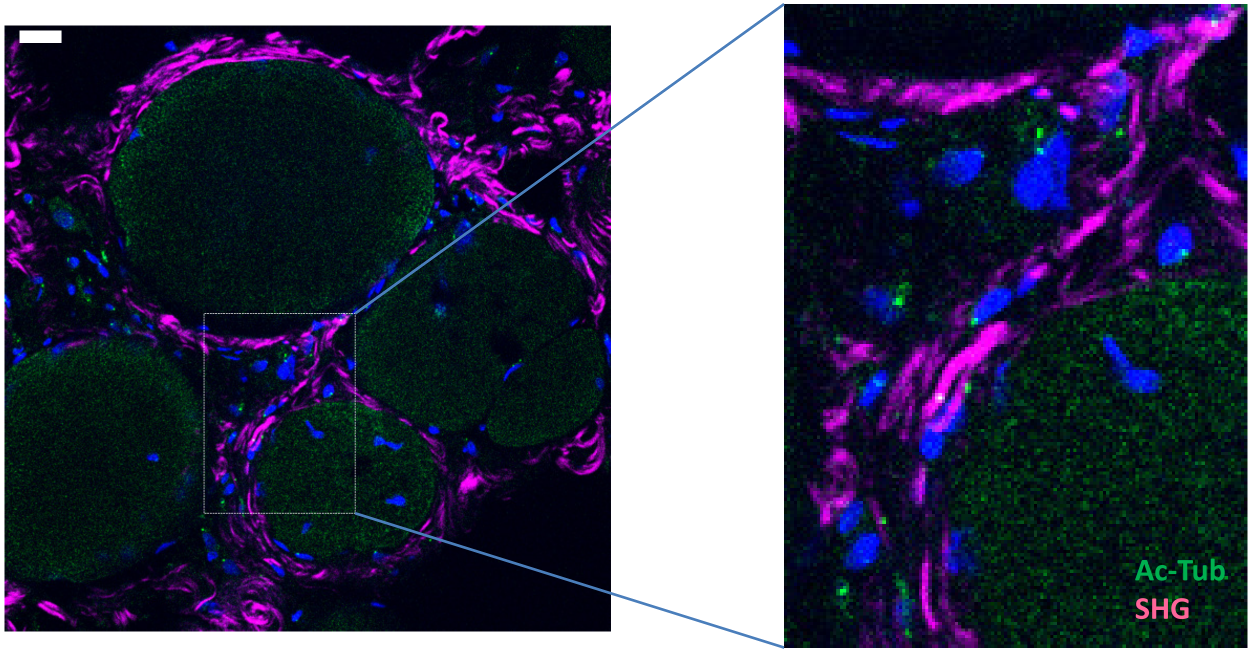

Supplementary Fig 1: Sections of paravertebral muscle from Duchenne myopathy patient were fixed and Ac-Tub (green) was revealed by immunocytochemistry. Collagen was revealed through second-harmonic generation imaging (SHG, magenta) and nuclei were stained with Hoechst33258 (blue). The white bar represents 50 µm.

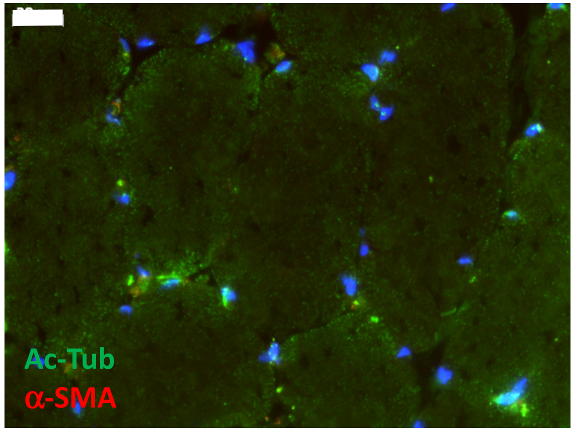

Supplementary Fig 2: Sections of muscle from healthy patient were fixed. Ac-Tub (in green) and α-SMA (red) were revealed by immunocytochemistry. The white bar represents 20 µm.

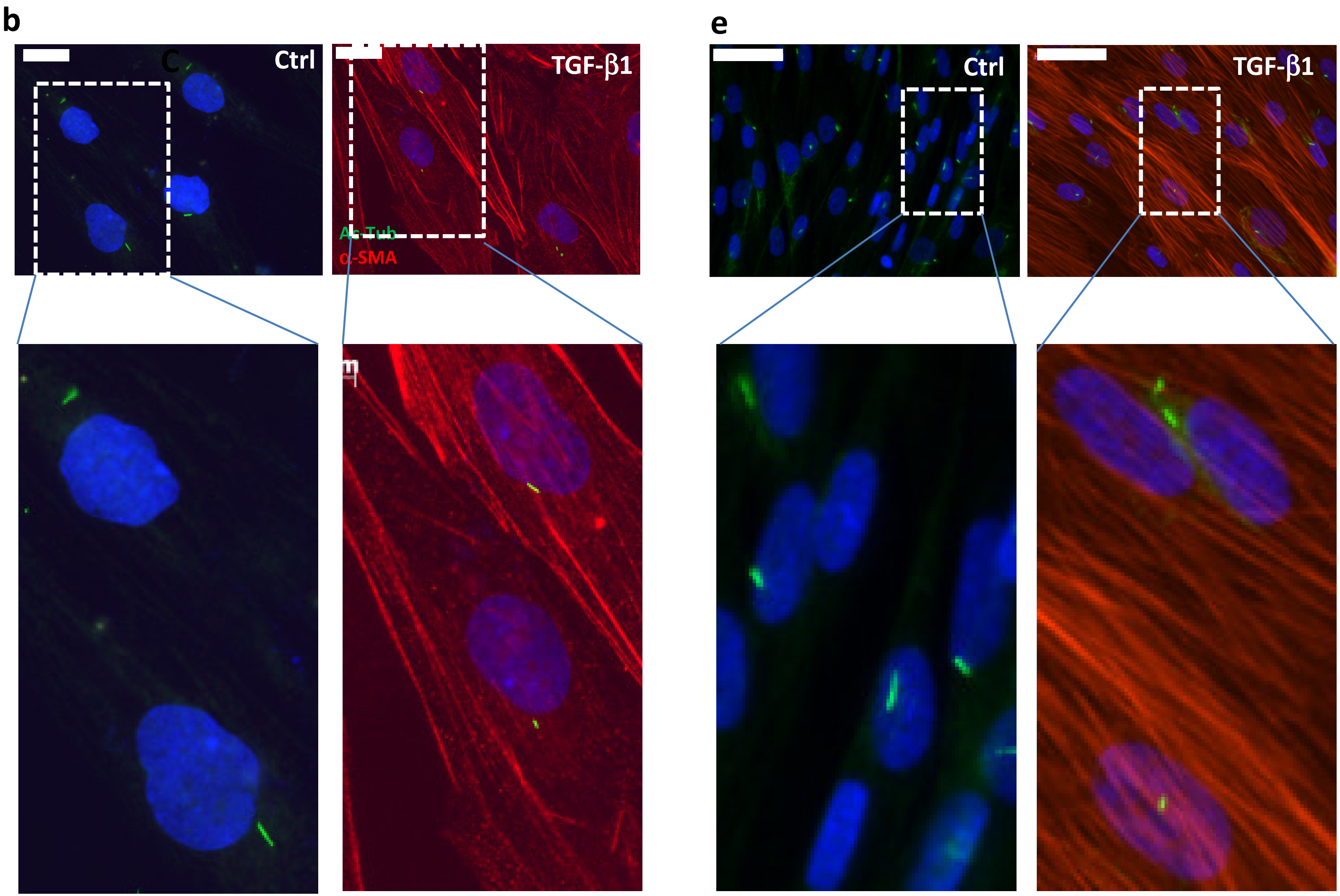

Supplementary Fig 3: This is an enlargement of panels b and e of Figure 1

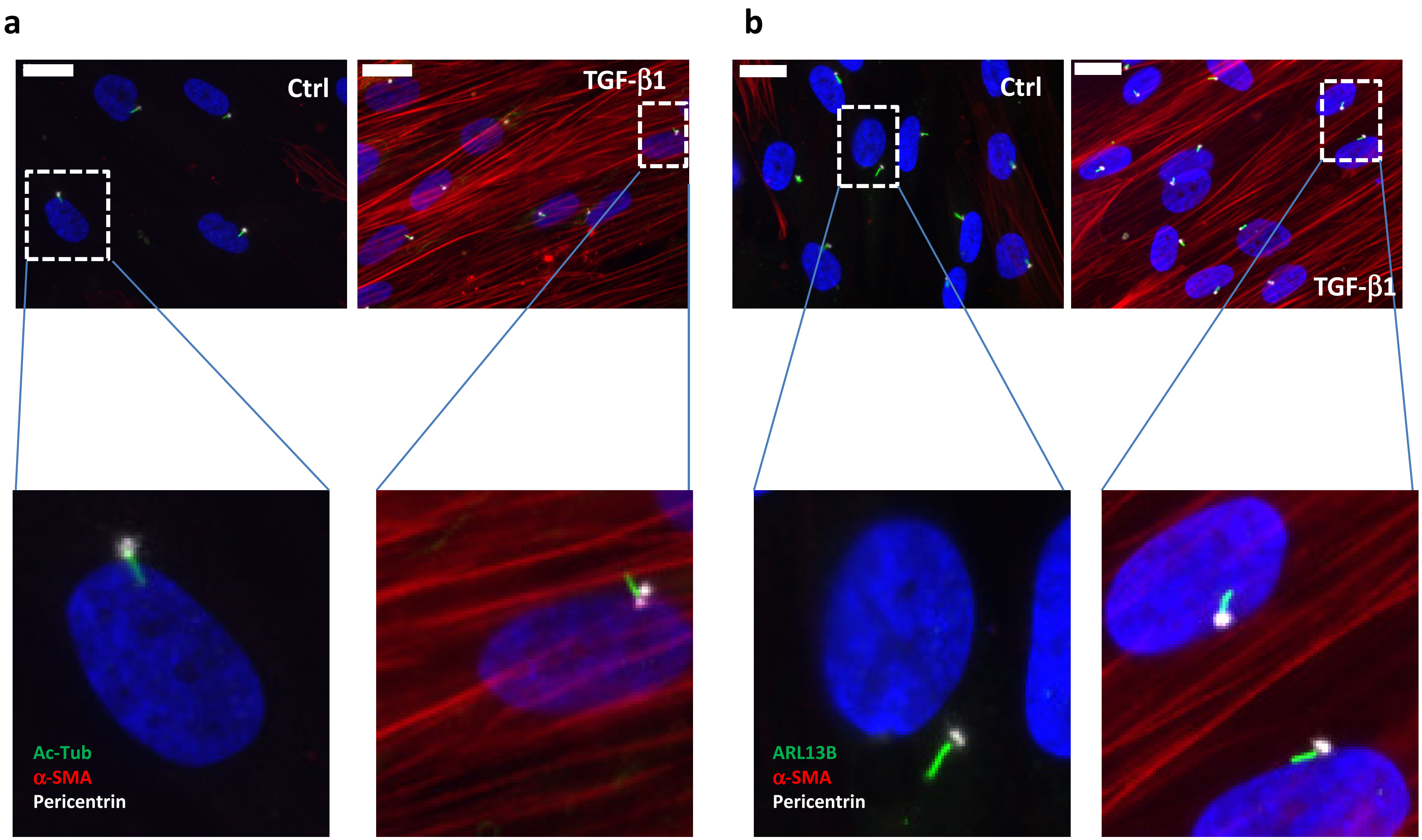

Supplementary Fig 4: aAPs were treated or not with 5 ng/ml of TGF-β1. In panel a) Ac-Tub (green), α-SMA (red) and pericentrin (gray) were revealed by immunocytochemistry. In panel b ARL13B (green), α-SMA (red) and pericentrin (gray) were labelled. The two dots corresponding to pericentrin labelling revealed the two centrioles of the basal body. Magnifications are provided bellow the pictures . The white bar represents 20 µm.

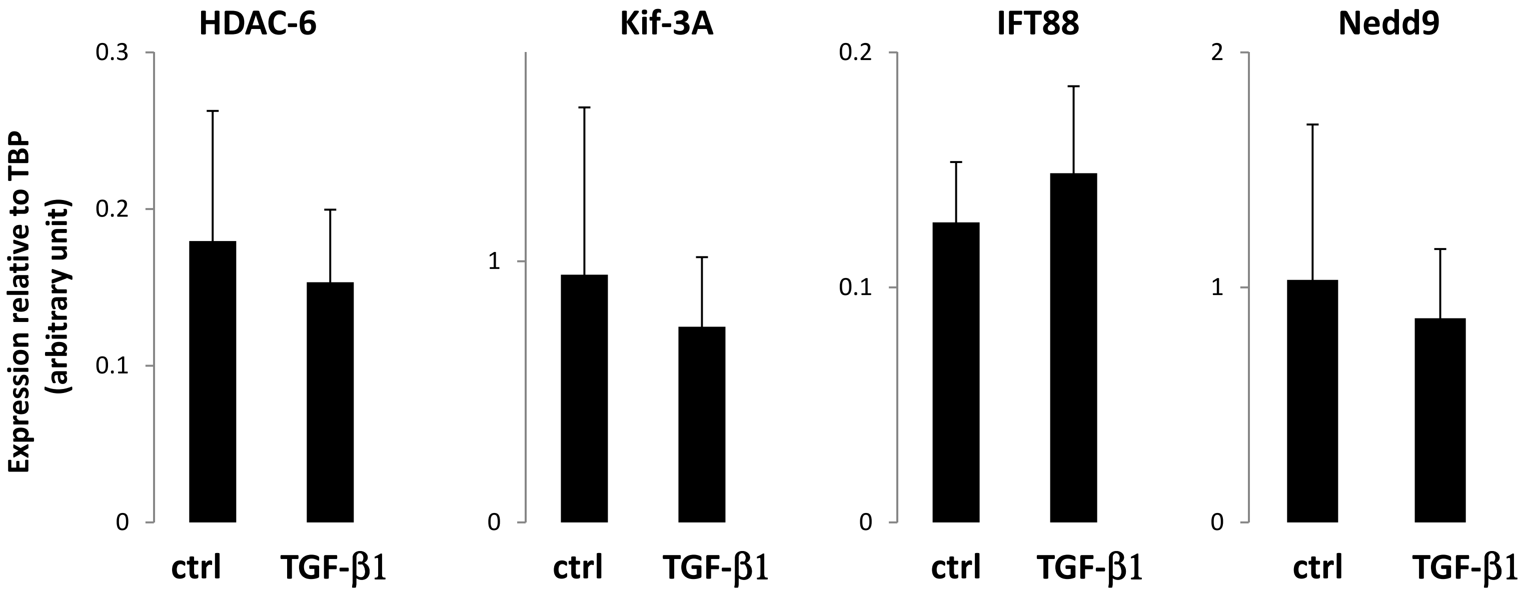

Supplementary Fig 5: aAPs were treated with TGF-β1 (5 ng/ml) for 3 days. RNAs were extracted and gene expression was assessed using quantitative RT-PCR.

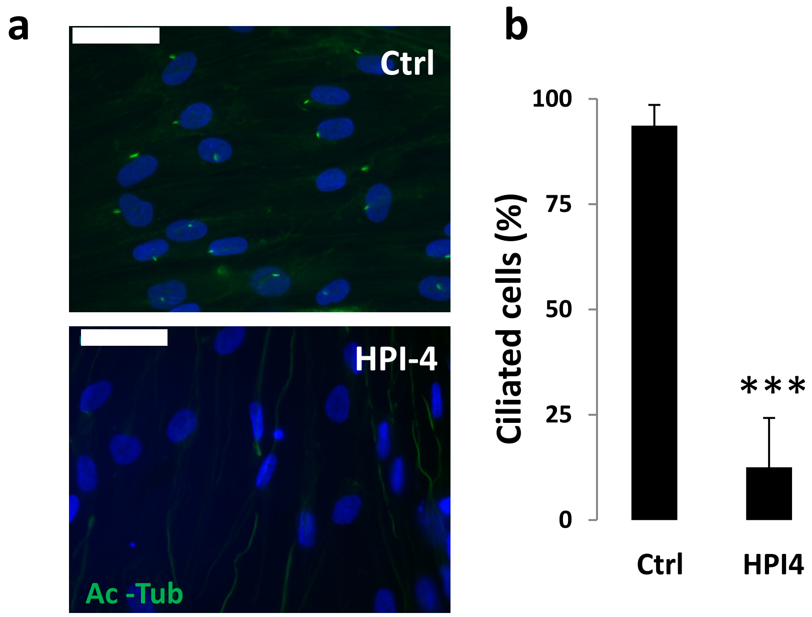

Supplementary Fig 6: a) aAPs were treated with or without HPI-4 (60 µM) for 24 h. Cells were fixed and Ac-Tub (in green) was revealed by immunocytochemistry. The white bar represents 50 µm. b) Percentages of ciliated cells were measured (***: $p<0.001$)

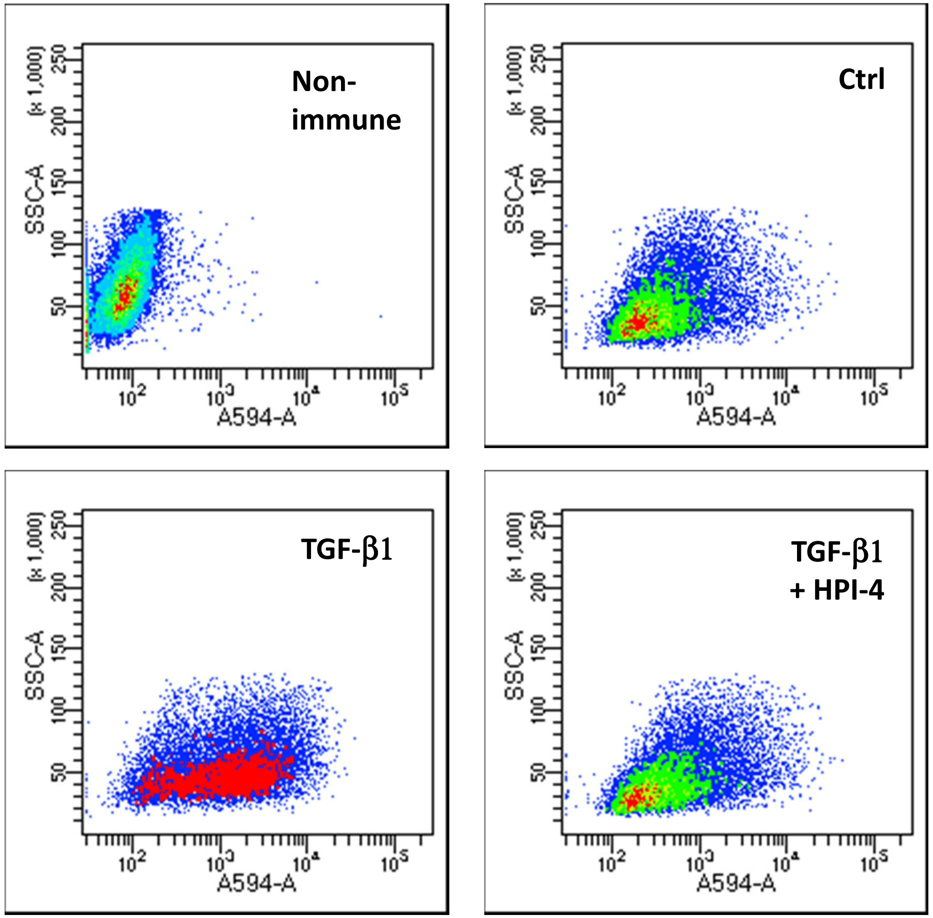

Supplementary Fig 7: dot plots corresponding to the FACS presented in Fig 2B and control performed with a non-immune primary antibody.

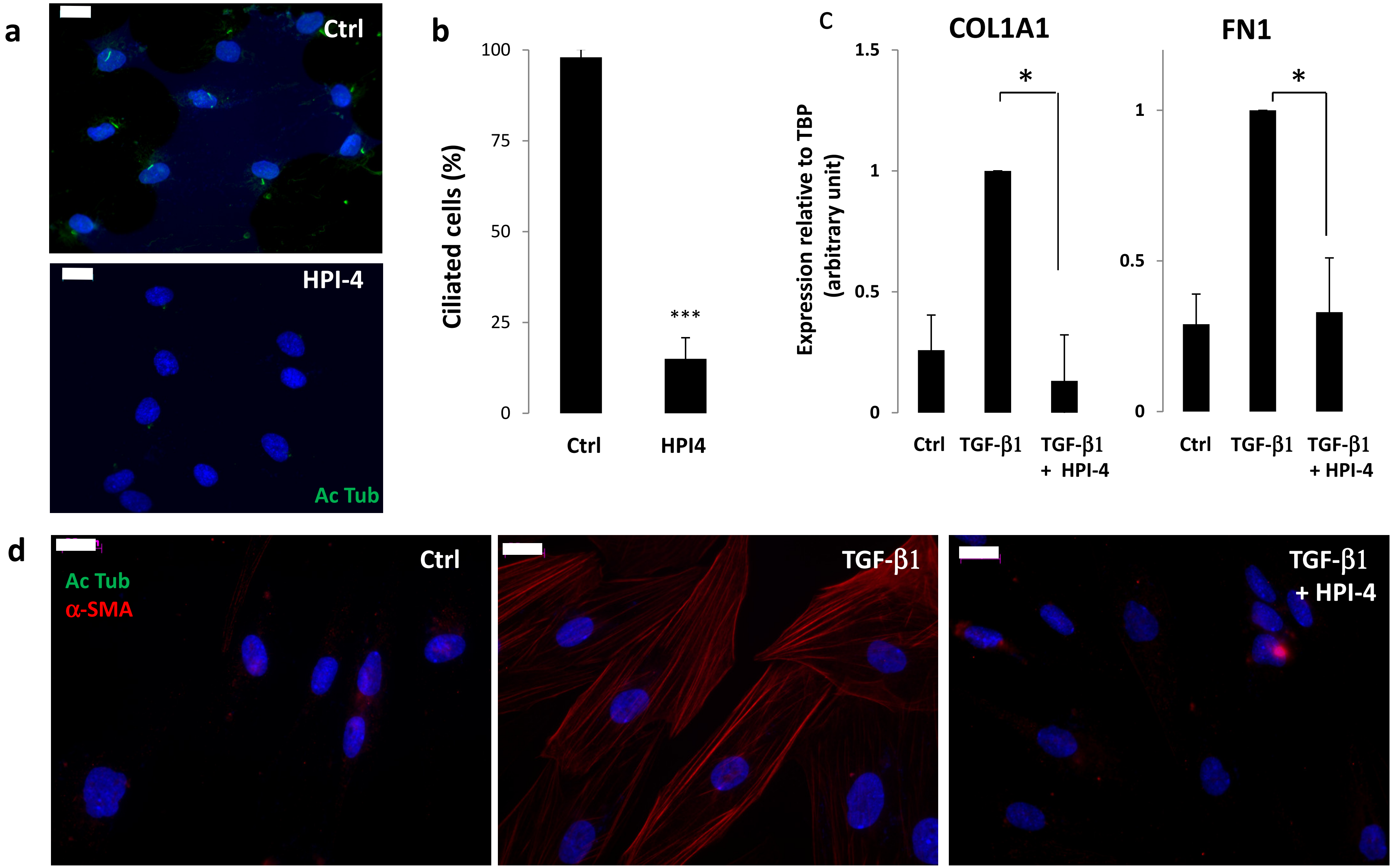

Supplementary Fig 8: A) mAPs were treated or not with HPI-4 for 24 h. Cells were fixed and Ac-Tub (in green) was revealed by immunocytochemistry. B) Percentages of ciliated cells were measured (n=4 ***$p<0.001$) C) mAPs were treated for 24 h with HPI-4. HPI-4 was removed and TGF-β1 added. After 5 days gene expression was assessed using quantitative RT-PCR (n=3 *: $p< 0.05$). D) Cells were fixed and Ac-Tub (in green) and α-SMA (red) were revealed by immunocytochemistry. The white bar represents 20 µm.

Original pictures from Fig 2d

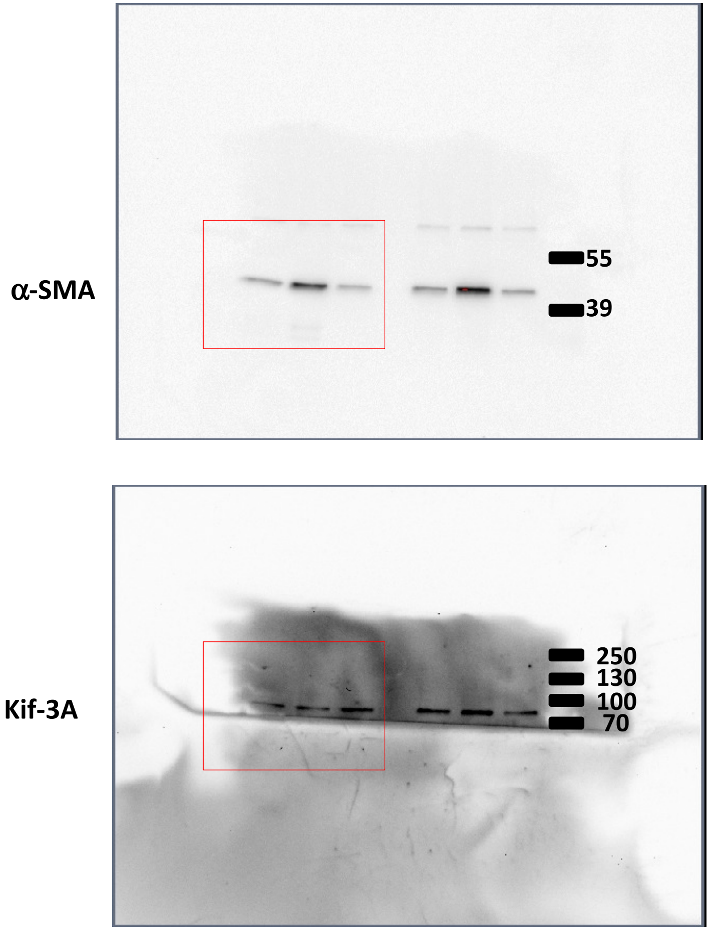

This figure and the following represent the whole field of the pictures of the Western blots taken by a Biorad Chemidoc XRS+ imaging system. The molecular weights (kd) and the antibodies used are indicated on the Western blots. In several experiments membranes were cut according to the molecular weights and probed with several antibodies (as indicated). In some experiments several membranes were exposed at the same time.

Original pictures from Fig 3a

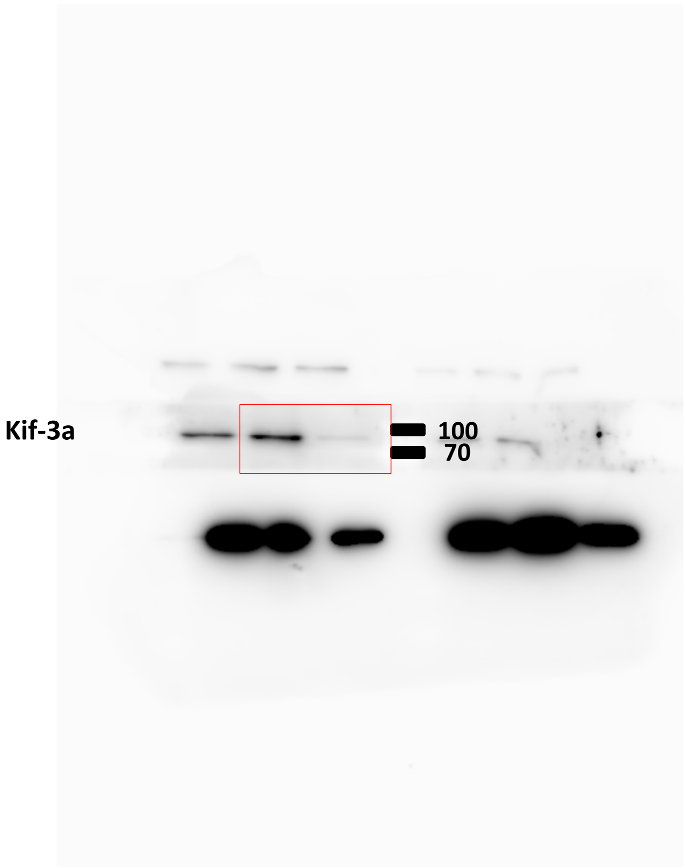

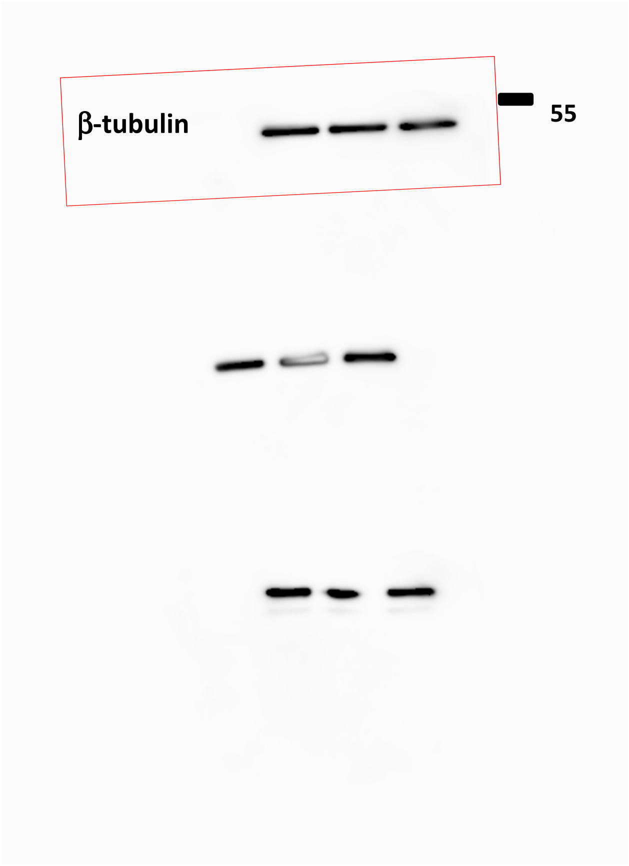

Original pictures from Fig 3e

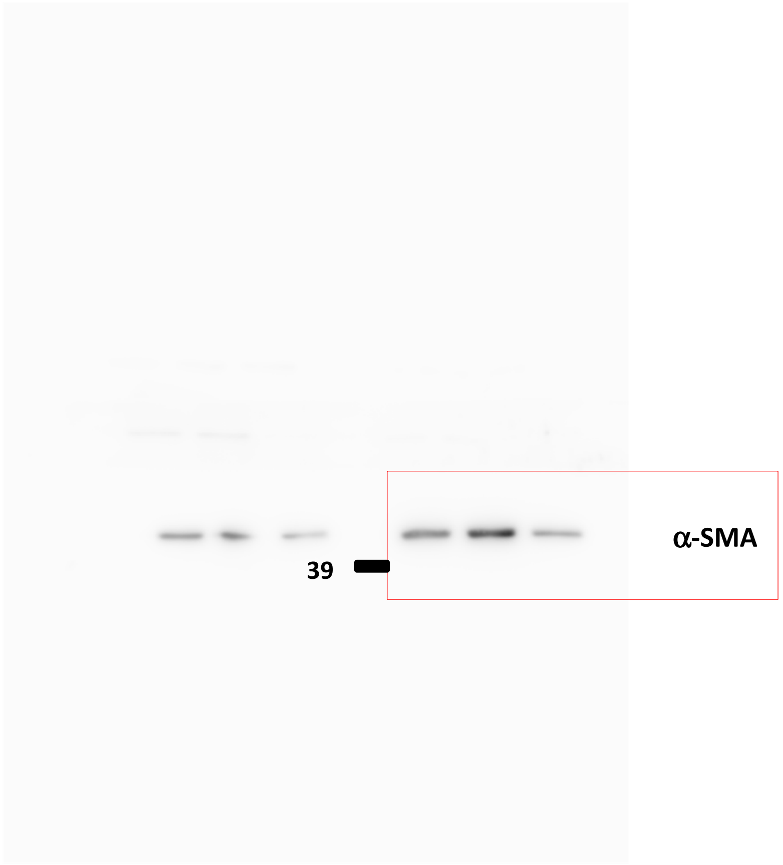

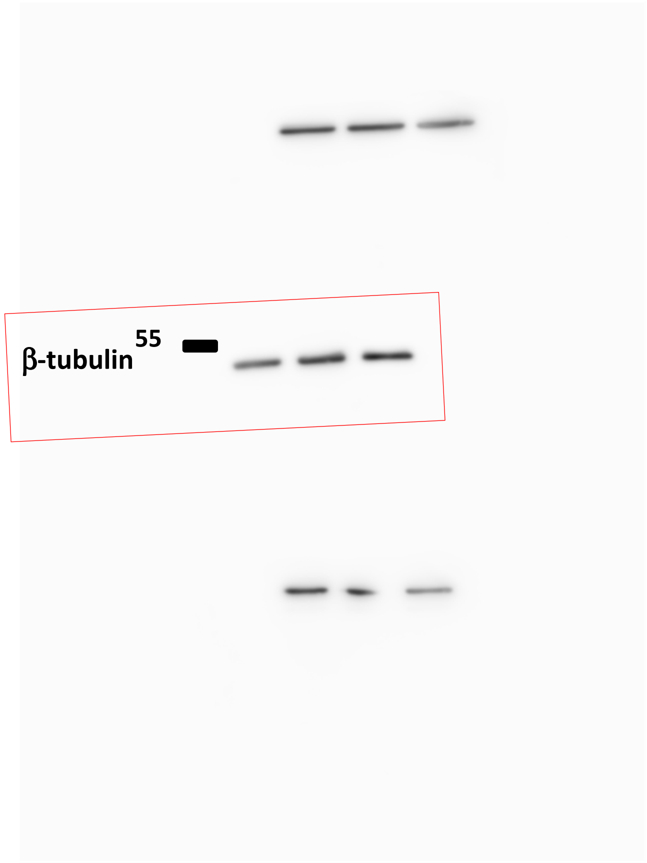

Original pictures from Fig 5a

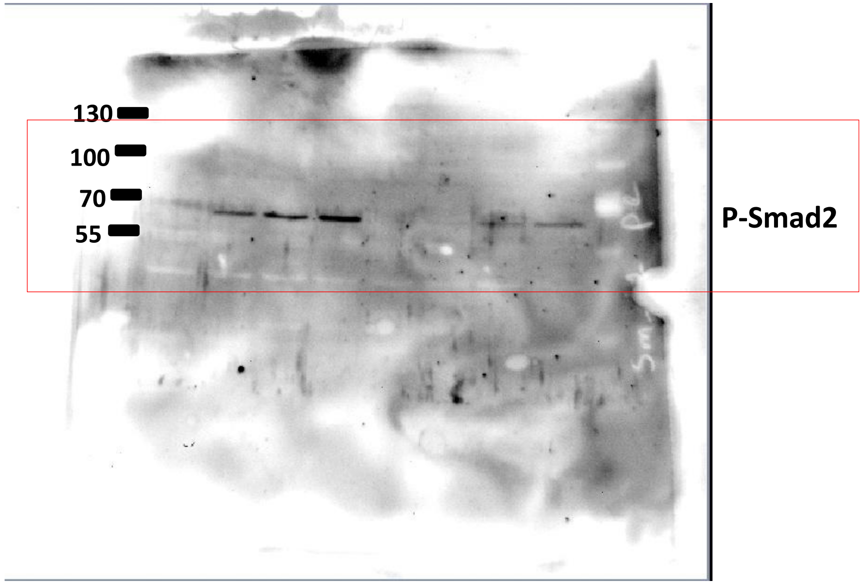

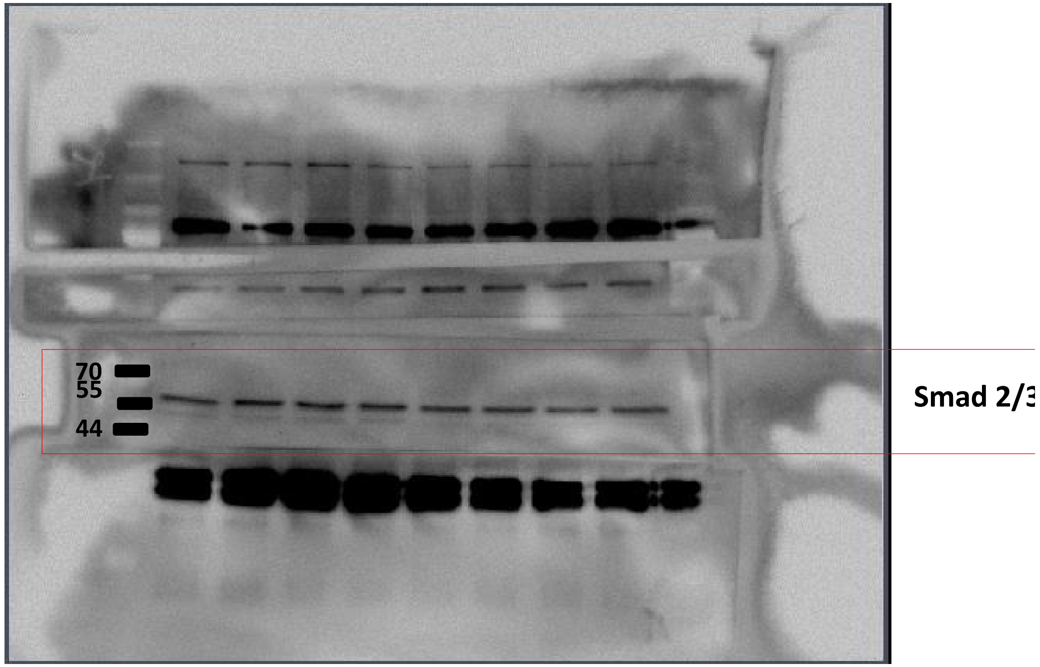

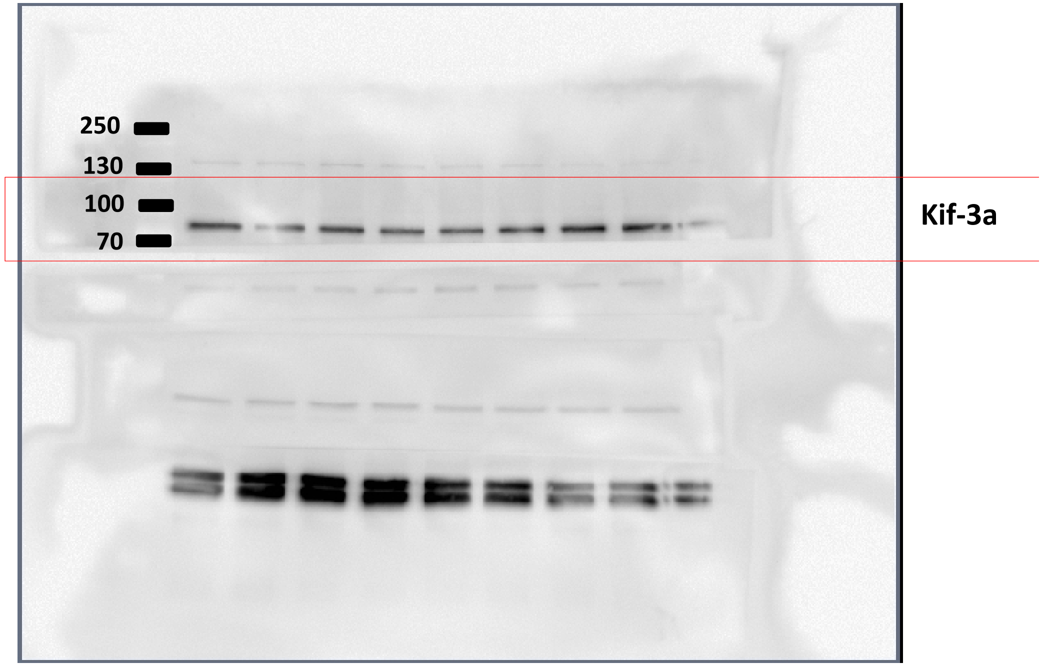

Original pictures from Fig 5b

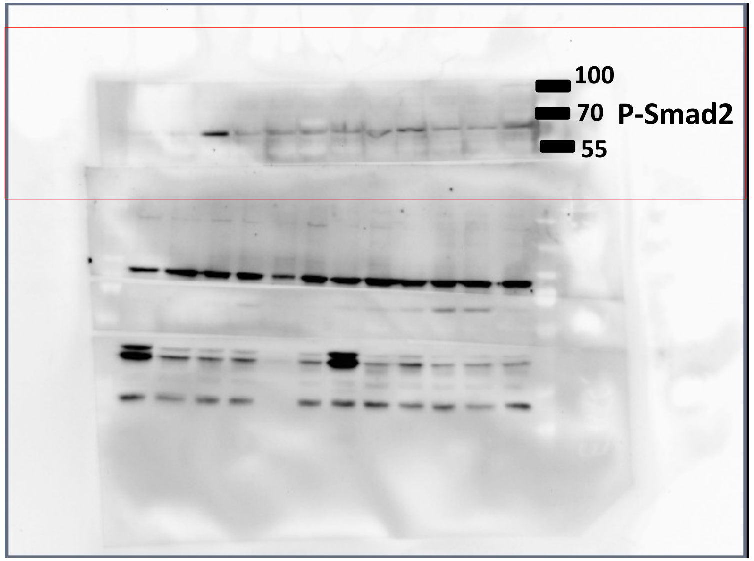

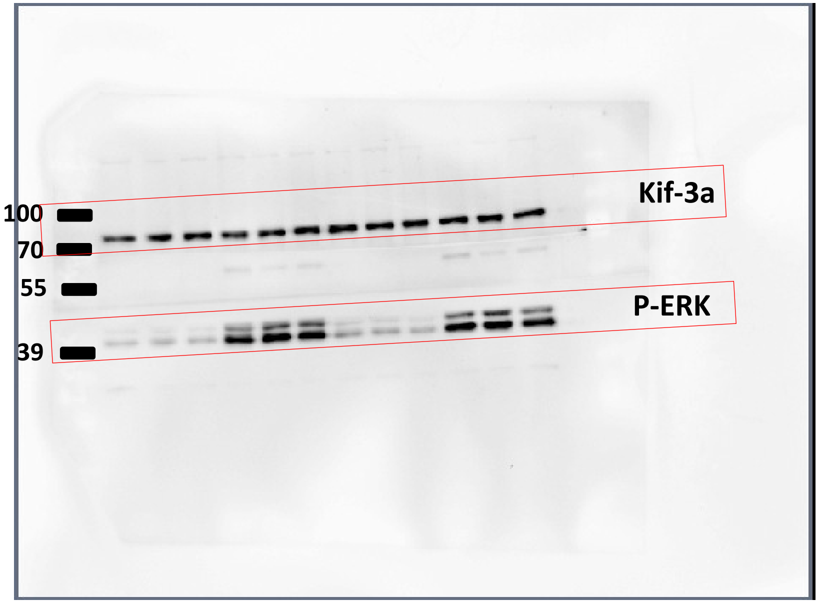

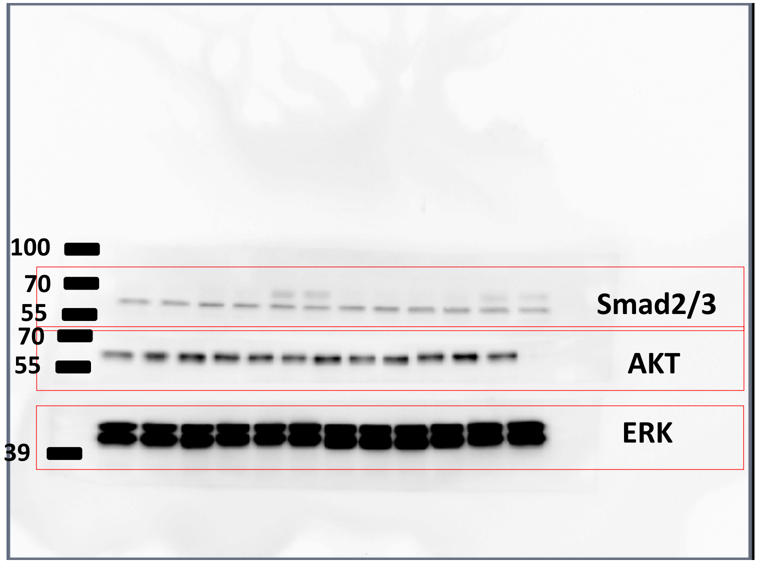

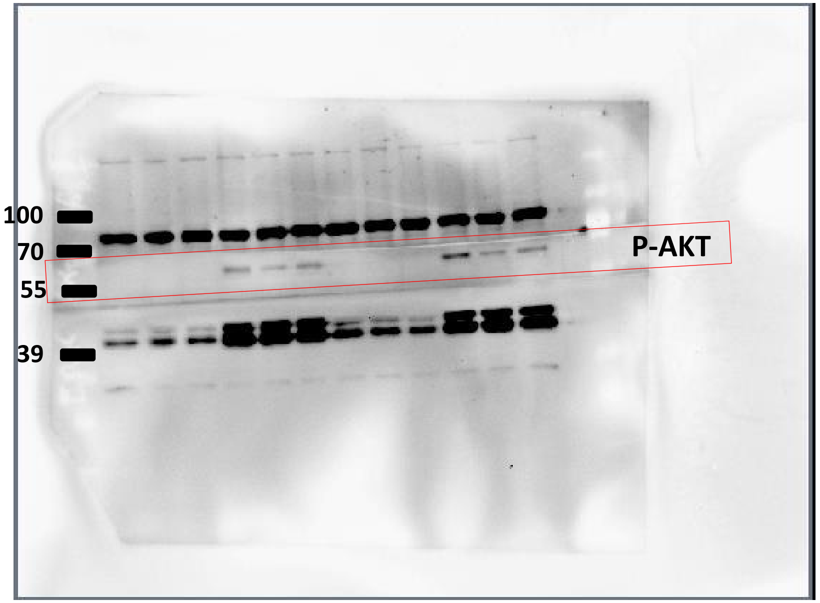

Original pictures from Fig 5c

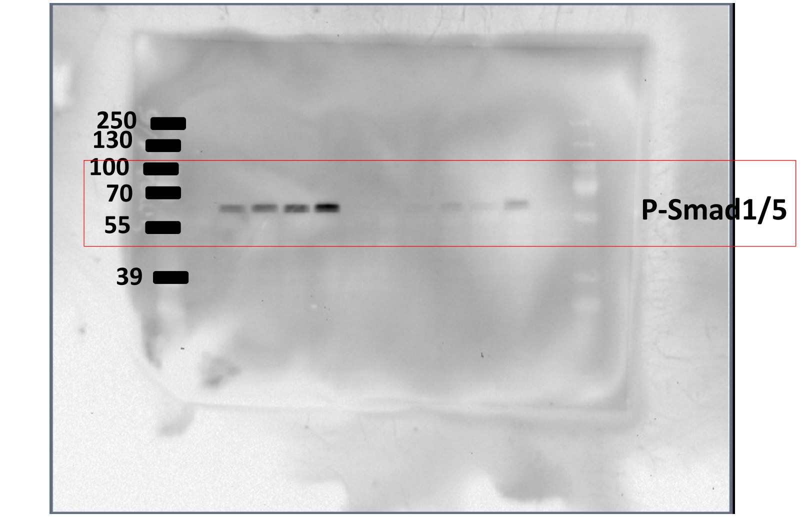

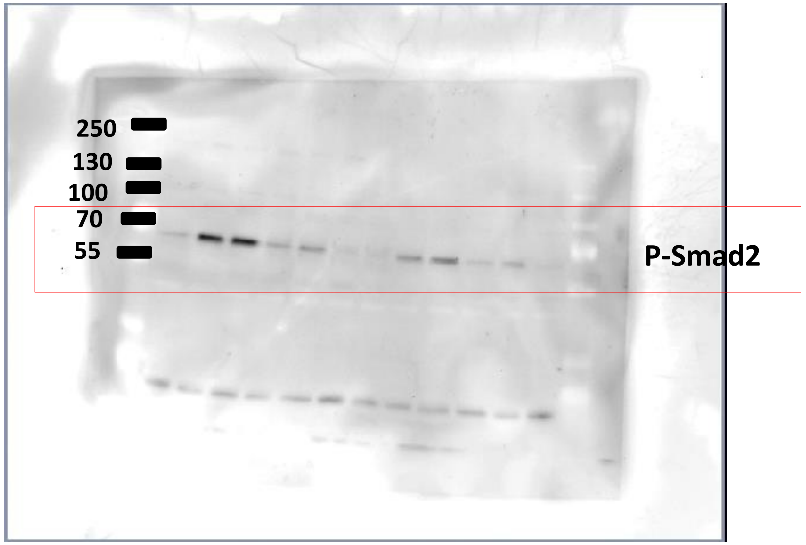

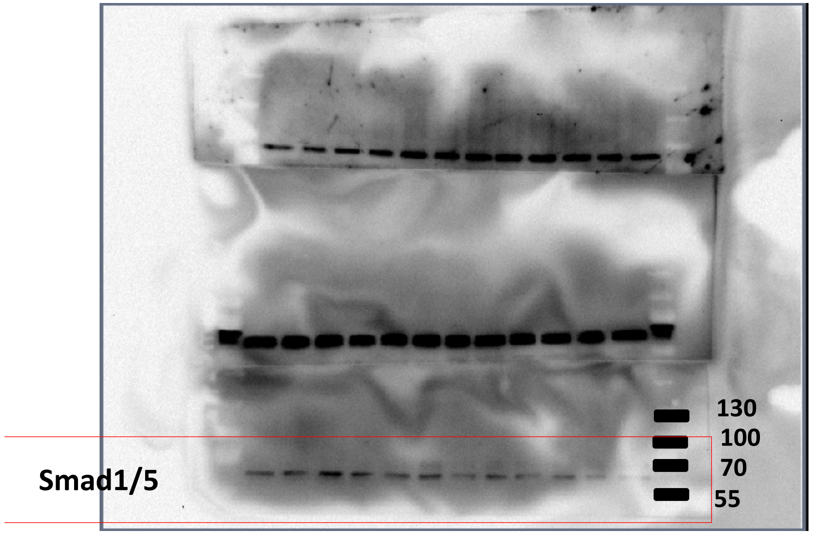

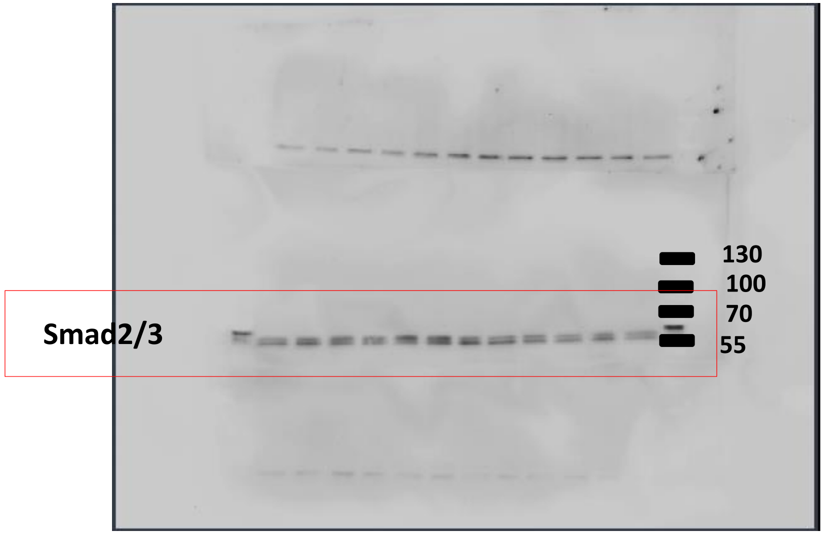

Original pictures from Fig 5c

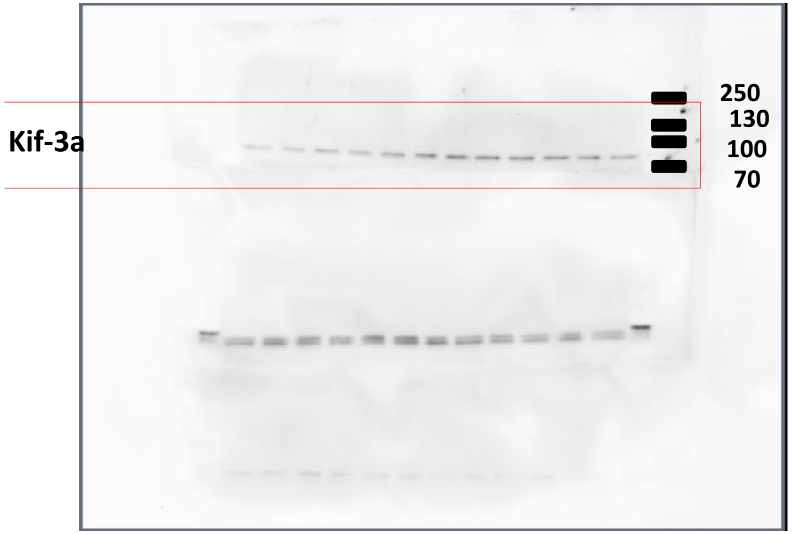

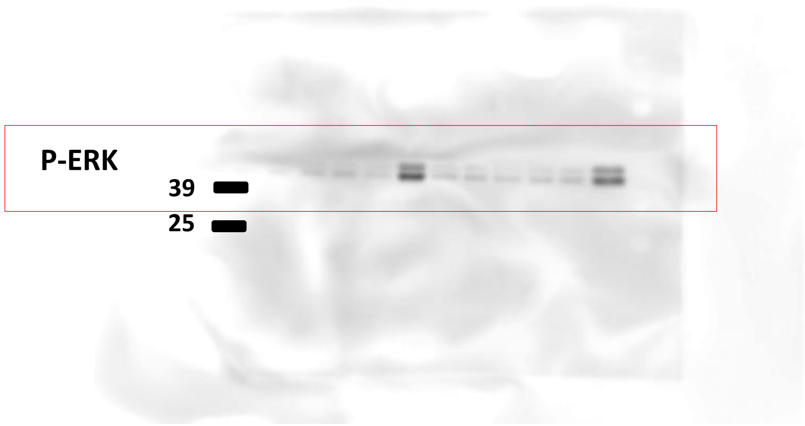

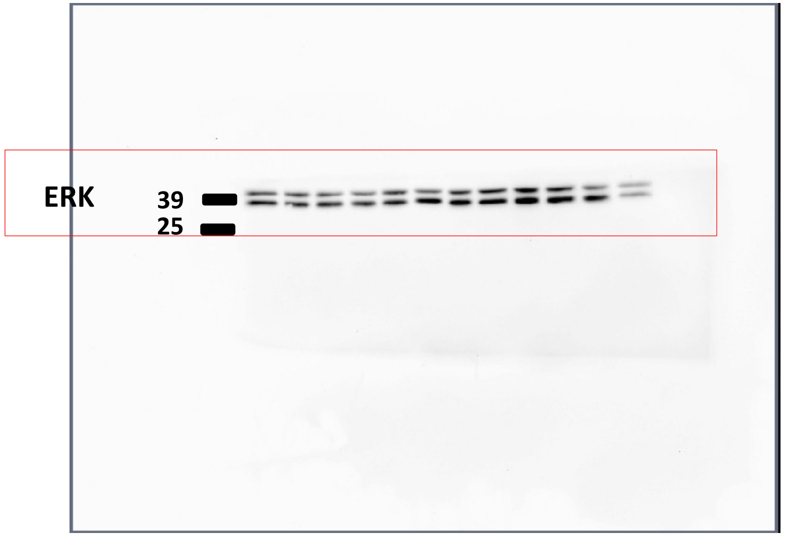

Supplement: Supplementary file 1 — Supplementary figures [file 41598_2017_15649_MOESM1_ESM.pdf]
